# Recruitment and retention strategies for improving representation in clinical research: A meta-synthesis

**DOI:** 10.1371/journal.pone.0322796

**Published:** 2025-06-23

**Authors:** Carson J. Peters, Joan M. Greve, Arvin Karbasi, Michelle Walker, Luyi Adesanya, Joyonna Gamble-George, Nicole Redmond, Brenda Adjei, Olufunmilola Olufemi

**Affiliations:** 1 Department of Behavioral and Community Health, School of Public Health, University of Maryland, College Park, Maryland, United States of America; 2 Small Business Innovation Research Development Center, National Cancer Institute, National Institutes of Health, Bethesda, Maryland, United States of America; 3 School of Medicine, Stanford University, Stanford, California, United States of America; 4 Department of Health Policy and Management, School of Public Health, University of Maryland, College Park, Maryland, United States of America; 5 School of Public Health, Loma Linda University, Loma Linda, California, United States of America; 6 College of Nursing, New York University, New York, New York, United States of America; 7 Division of Cardiovascular Sciences, National Heart, Lung, and Blood Institute, National Institutes of Health, Bethesda, Maryland, United States of America; 8 Office of the Associate Director of the Healthcare Delivery Research Program, National Cancer Institute, National Institutes of Health, Bethesda, Maryland, United States of America; 9 Office of Technology Transfer, National Institute of Neurological Disorders and Stroke, National Institutes of Health, Bethesda, Maryland, United States of America; The University of Alabama, UNITED STATES OF AMERICA

## Abstract

**Objective:**

To identify innovative strategies that may increase recruitment and/or retention of groups less represented in chronic disease clinical research.

**Methods:**

A systematic review was conducted. Inclusion criteria were: (a) NIH-defined racial and ethnic minority groups and clinical research; (b) evidence-based, clinical research recruitment and/or retention strategies involving the leading causes of mortality and morbidity in the United States; (c) conducted in the United States; and (d) qualitative design. Data exploring the strategies were extracted and thematically analyzed.

**Results:**

Twenty-seven studies were included. Studies focused on cancer (70%), recruitment (93%), and perspectives from clinicians (63%). The most referenced strategies were education (44%), communication (48%), and community-based participatory research (63%). Critical themes include empowerment, transparency, trust, and sustainability.

**Conclusions:**

Strategies must prioritize the community and be implemented sustainably, where cultural humility and community-based participatory research are foundational.

## Introduction

The goal of clinical research is to find ways to prevent, diagnose, and treat disease to improve the overall health and quality of life for all people more effectively. Despite significant investments in clinical research, the differences in health status and health care for many groups in the United States has not been mitigated [1]. With the latest United States (U.S.) census in 2020 [2], the impact of population health challenges will grow exponentially without action.

For nearly 30 years, federal law, National Institutes of Health (NIH) policies, and Food and Drug Administration (FDA) guidance [3,4] have sought to achieve optimized health by emphasizing ethical engagement and representation of all people in clinical research. With less than 15% of clinical trials reporting outcomes by race or ethnicity [5,6] and many investigators still struggling to ensure appropriate representation [7,8], it appears more impactful efforts are needed. In addition, juxtaposing these results is evidence suggesting that despite potential hindrances to research participation such as trust, time, and financial concerns, groups less represented in clinical research indeed “are willing to engage in clinical research” [9–11].

To explore this dissonance of the lack of representation in clinical research, despite the willingness of these groups to participate, we conducted a systematic review to identify evidence-based strategies used to improve recruitment and retention of all groups in clinical research. To our knowledge, this systematic review is among the first designed to qualitatively assess strategies across less represented groups and chronic diseases. Since there is “substantial quantitative data demonstrating the problem of underrepresentation” in clinical research, we sought to achieve a primary objective set forth in Chapter 5 of the recent National Academies of Science, Engineering, and Medicine (NASEM) report entitled *Improving Representation in Clinical Trials and Research: Building Research Equity for Women and Underrepresented Groups* [12] and provide evidence-based facilitators of appropriate representation in clinical research. The studies included in our review are primarily from the perspective of individuals across the continuum of clinical research, i.e., participants, caregivers, clinicians, community leaders, and medical center leaders, which complement the NASEM report’s focus on research teams. The results were thematically analyzed to develop a conceptual model from which actionable recommendations emanate. These results align with our unpublished qualitative analysis of NIH-funded clinical studies, for strategies that improved the participation of less represented populations in clinical research. The goal of this review is to provide clinical research teams with strategies that enhance the recruitment and retention of populations in clinical research.

## Methods

We adhered to and adapted the Enhancing Transparency in Reporting the Synthesis of Qualitative Research (ENTREQ) guidelines [13], Enhancing the Quality and Transparency of Health Research (EQUATOR) guidelines [14], and The Preferred Reporting Items for Systematic Reviews and Meta-Analyses (PRISMA) checklist to report this completed systematic review and meta-synthesis [15].

### Eligibility criteria

Inclusion criteria are detailed in S1 Table. Briefly, we included studies with qualitative or mixed methods research designs, were conducted in the U.S., published in English or Spanish in a peer-reviewed journal between 2009 and 2024, and that demonstrated evidence-based recruitment and/or retention strategies for clinical research focused on the leading causes of morbidity and mortality in the U.S. as determined by the Centers for Disease Control and Prevention (CDC) [16]. Studies spanning ten years from 2009 were initially selected; this was later updated to include fifteen years from 2009, to reflect the increasing significance and importance of this work during this period. Eligible studies targeted minorities defined by NIH [17]. Studies included all ages and used the NIH definition of clinical research [18].

### Information sources and search strategy

The literature search strategy was developed in collaboration with the review team and trained biomedical librarians (NT and AL) at the National Institutes of Health (NIH). The search strategy was created using a combination of text words and the controlled vocabulary terms in the following databases: (PubMed (MeSH) Medical Subject Headings, Embase – EMTREE, and CINAHL subject headings. The search was refined using an iterative process and finalized by the review team members and librarians. For each search strategy the search terms included these text words and controlled vocabulary when available: underrepresented, minority, racial and ethnic groups, clinical research and disparities. The following databases were searched: PubMed (National Library of Medicine), Embase (Elsevier), CINAHL Plus (Cumulative Index to Nursing and Allied Health Literature - EBSCOhost), and Web of Science Core Collection (Clarivate Analytics). The following limits were applied using the filters available in each database. The search was limited to human studies only and studies conducted in the United States. The final search strategy can be found in the S2 Appendix.

### Selection process

Covidence systematic review software (Veritas Health Innovation, Melbourne, Australia; www.covidence.org) imported studies and automatically excluded duplicates. All stages of the screening, data extraction, and quality assessment were independently conducted by members of the review team (CJP, JMG, AK, MW, LA, and JGG). The review team was composed of six members.

The review team first screened titles and abstracts to identify studies that met the inclusion criteria. Next, the full texts of studies included during title and abstract were screened using the same eligibility criteria. Each article was screened by two reviewers and conflicts between reviewers were resolved by consensus discussion with the review team.

### Data collection process & data items

Data from each included study was collected by two reviewers using Covidence. The following outcomes of interest were extracted: focus on recruitment, retention, or both; and a description of the evidence-based strategies. We extracted data on study characteristics including year of publication and condition of interest, including sub-type for cancer. Additionally, we extracted characteristics including race and ethnicity, sex if applicable, geography (urban or rural), and role in clinical research (e.g., participant, clinician (i.e., medical or research staff), community leader, etc.).

### Study risk of bias assessment

For the quality assessment, we evaluated the following domains: (a) role of the researcher; (b) sampling method; (c) data collection method; and (d) analysis method, which were identified as *all criteria met* or *criteria partially met*. We followed the adapted guidelines and conceptual domains of the Critical Appraisal Skills Programme (CASP) quality assessment tool [13,19,20] to assess the quality of studies. Two reviewers assessed the risk of bias for each included study and resolved disagreements by consensus discussion with the review team.

### Synthesis methods

A thematic synthesis was operationalized for the data analysis [13,21] where we analyzed the findings and developed inductive and deductive codes [22] using qualitative synthesis methodologies [22,23] and established guidelines [24–26]. The thematic synthesis utilized an iterative process grounded in qualitative thematic analysis methodologies [22,23]. We initially developed a deductive coding scheme, focusing on direct meaning and content that highlighted evidence-based strategies and direct quotations from study participants. The team discussed and created the codebook, and then each code was defined. Discrepancies or additional deductive codes were added and discussed by the team for consensus. Each study was coded independently by reviewers. Inductive codes were later added to describe high level interpretation and themes. Both deductive and inductive codes existed in our codebook using this iterative process. Further analysis was conducted where strategies and themes were summarized into a conceptual model emphasizing key elements for recruitment and retention of racial and ethnic groups historically underrepresented in clinical research [21,27].

## Results

**Fig 1:**
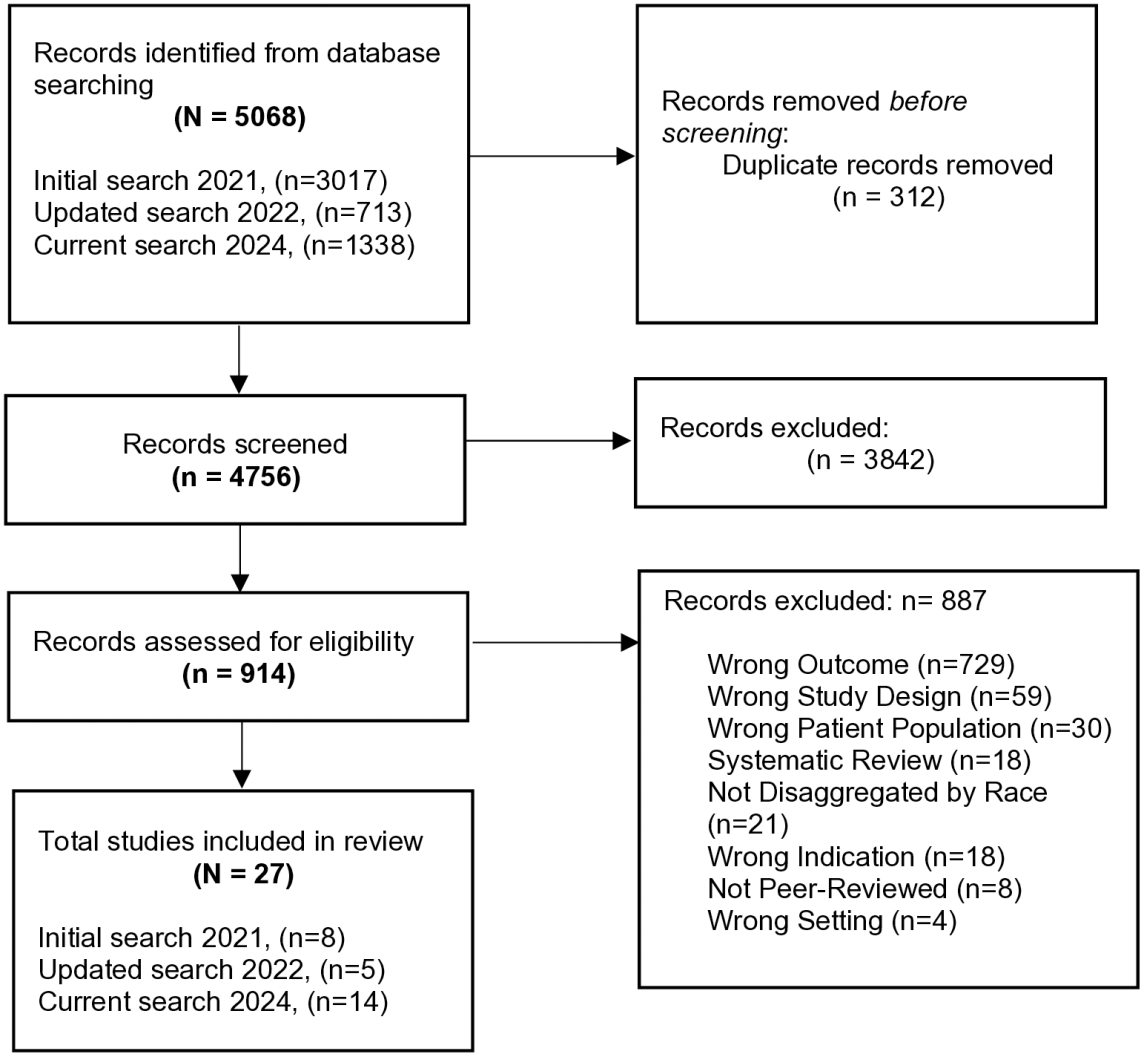
Flowchart of Study Selection. Wrong outcome = study does not mention evidence-based strategies; Wrong Study Design = study is not qualitative nor mixed methods design; Wrong patient population = study not focused on race and ethnic groups of interest; Systematic review = study is a systematic review; Not disaggregated by race = unable to determine which outcomes were associated with which race and ethnic group; Wrong indication = not one of the leading causes of mortality and morbidity in the U.S.; Not peer-reviewed, e.g., commentary; Wrong setting = study is not based in U.S.

Our initial literature review focused on studies that were published between 2009–2020. Subsequent updated searches were performed in 2022 and 2024, to provide a comprehensive update of literature on this topic. Of the total 5,068 studies identified for initial title and abstract screening, 914 studies underwent full text review with 27 studies proceeding to data extraction and quality assessment (Fig 1, [28–53]).

S2 Table describes the characteristics of each study included in this meta-synthesis systematic review while Table 1 summarizes the studies in aggregate, separating inclusion criteria from other data extracted during the review. Twenty-five studies implemented recruitment strategies, and two studies implemented both recruitment and retention strategies. Eleven studies focused primarily on Black/African American communities, with four studies involving Latino/Hispanic, two studies involving Asian and one study involving Native Hawaiian/ Other Pacific Islander communities and eleven studies focused on People of Color (POC). No studies focused on American Indian/Alaska Native communities. Nineteen studies (70%) focused on cancer (breast, hematologic, lymphoma, multiple cancers, or not reported), six (22%) focused on Alzheimer’s and dementia, one (3.7%) study focused on kidney disease and one (3.7%) study focused on heart disease. No studies included chronic respiratory diseases, diabetes, pneumonia and influenza, or stroke. Although not used as inclusion criteria for this systematic review, we extracted data related to sex, geography, and with whom the study collected strategies from (sources role in clinical research). Sex was acknowledged in ~40% of the studies, with eleven studies reporting sex of the clinical research participant and/or the person’s role in clinical research. No studies included persons under 18 years old. While nearly 80% of studies reported on geography, no studies solely focused on rural locales. All told, the studies included in this systematic review reflect strategies from individuals whose roles in clinical research were participants, participant networks (i.e., study partner, caregivers), clinicians (i.e., medical staff, healthcare providers, research staff), medical center leadership, recruiters, and community leaders. The characteristics of the study were based on percentages, where two studies included multiple populations [28,42] five studies reported on sex across multiple groups [38,45–47,51]; and seven studies reported multiple roles in clinical research [31,35,38,39,45–47,51]. Studies reporting multiple groups are counted for separately in Table 1.

**Table 1:** Summary of included studies for meta-synthesis of evidence-based strategies (N = 27).

Study Purpose (inclusion criteria)	Number of Studies	Percent
Recruitment	25	92.6%
Retention	0	0
Both	2	7.4%
**Race and Ethnicity (inclusion criteria)**		
American Indian/Alaska Native	0	0
Asian	2	7.4%
Black/African American	11	40.7%
Native Hawaiian/ Pacific Islander	1	3.7%
Latinx/Hispanic	4	14.8%
POC	11	40.7%
Condition of Interest (inclusion criteria)*		
Alzheimer’s/Dementia	6	22.0%
Heart Disease	1	3.7%
Kidney Disease	1	3.7%
Cancer	19	70.3%
Breast	3	11.1%
Hematologic	1	3.7%
Lymphoma	1	3.7%
Melanoma	1	3.7%
Multiple	2	7.4%
Not reported	11	40.7%
**Sex**		
Female, as study criteria	11	40.7%
Male, as study criteria	5	18.5%
Not reported for the source, not part of study criteria	17	62.9%
**Geography**		
Urban	4	14.8%
Rural	0	0
Both	17	62.9%
Not reported	6	22.2%
**Role in clinical research**		
Clinicians (Research Staff, Medical Staff/Providers)	17	62.9%
Community Leaders	1	3.7%
Participant Networks (Caregivers, Study Partner)	3	11.1%
Participants	11	40.7%
Medical Center Recruiters	4 1	14.8% 3.7%

*Chronic respiratory diseases, diabetes, pneumonia and influenza, and stroke are not listed because no studies were included for these indications. Data in Ridley-Merriweather included two racial and ethnic groups (Asian, Latino). Data in TaPark included two racial groups (Asian, Native Hawaiian/ Pacific Islander). In both studies, each racial and ethnic group was counted separately. Five studies reported inclusion of sex across multiple categories, which were also counted separately. Seven studies reported multiple roles in clinical research are counted for separately.

Twelve studies met all quality criteria, while fifteen did not meet all criteria (S2 Table). The most common reason for not meeting all criteria was related to the sampling method and specifically studies not disaggregating strategies by race and ethnic group, i.e., grouping all individuals into POC.

Table 2 describes the deductive codes derived from the direct meaning and content of the studies included in this meta-synthesis systematic review, along with a summary definition, a representative quote, and a listing of the studies reflecting that particular strategy. S3 Table summarizes the frequency the strategies were observed across the included studies, with sub-groupings where appropriate. The most frequently utilized strategies were education (44%), communication (48%), and community-based participatory research (63%). Cultural humility was reflected in nearly 40% of studies. Strategies reflecting relationships (awareness, language, patient navigation, patient-provider relationship, social networks, technology) were evident in nearly one-quarter of studies. And, finally systems, and training were noted in ~20% of studies.

**Table 2:** Deductive coding from meta-synthesis of studies on recruitment and retention strategies for groups less represented in clinical research.

Recruitment or Retention Strategy	Summary definition	Quote	Study
**Awareness**	Disseminates information about clinical research and/or the benefit of research through advertisements and sharing information.	...I *purposefully put on wellness fairs just so that the information can get to the population *that's* here, because I know the information is out there, but I know that unless we bring it to them, they—it will never be advertised, it will never come, you will never know it exists” (African American, Participant;* Lincoln 2021, p.591)	Currier 2023, Fink 2023, Frierson 2019, Haynes-Maslow 2014, Lincoln 2021, Niranjan 2021, Portacolone 2020, Regnante 2020
**Community-Based Participatory Research**	Uses community-based organizations with community-based leadership; conducts research at community spaces or other existing spaces that hold value in the community like churches; establishes community partnerships; involves community leaders; implements community engagement; demonstrates commitment to the community; ensures co-design/creation of clinical research with the community throughout research process; shares feedback at all stages of design and development, and post-study; centers community voices and input.	*“Don’t wait ’til they come to you. Educate and build trust” (Leader;* Niranjan 2021, p.670)	Alvarado 2023, An 2023, Aranda 2023, Crabbe 2023, Currier 2023, Dance 2021, Frierson 2019, Hartley-Brown 2024, Haynes-Maslow 2014, Niranjan 2021, Portacolone 2020, Regnante 2020, Rivers 2019, Schatz 2023, Stockdill 2023, TaPark 2023
**Clear and Consistent Communication**	Illustrates clear and consistent communication including information sharing about the clinical research and narrative-based (i.e., storytelling) techniques about benefits, risks, research, etc.	*“… (I) want to know as much information as possible, even beyond… the informed consent” (Asian, Participant, Female;* Ridley-Merriweather 2022, p.6)	An 2023, Aranda 2023, Dance 2021, Haynes-Maslow 2014, Hernandez 2021, Joseph 2009, Legor 2023, Mesa 2023, Niranjan 2021, Portacolone 2020, Ridley-Merriweather 2022, Robinson 2020, Stockdill 2023
**Cultural Humility**	Ensures cultural humility including, but not limited to, cultural sensitivity, cultural competence, identity matching and/or adapts intervention to target audience.	*“That is the key, [not] trusting someone that doesn’t look like you, or doesn’t have an understanding of who you are.” (African American, Participant;* Lincoln 2021, p.593).	Alvarado 2023, Aranda 2023, Fink 2023, Frierson 2019, Hartley-Brown 2024, Legor 2023, Lincoln 2021, Niranjan 2019, Ridley-Merriweather 2022, Portacolone 2020
**Education**	Educates potential participants about clinical research, disease, risks, and/or about the advantages of research through programs and materials (i.e., flyer, poster, pamphlets).	*I think education, informing the patients, empowering the patients I think through churches, through whatever access or community mechanism you have may help.…. (Research Staff,* Niranjan 2021, p.669)	Dance 2021, Fink 2023, Frierson 2019, Hartley-Brown 2024, Hernandez 2021, Lincoln 2021, Niranjan 2021, Portacolone 2020, Ridley-Merriweather 2022, Rivers 2019, Robinson 2020, Schatz 2023
**Language**	Provides resources, materials and translation in multiple languages and uses clear verbiage catering to varying language proficiency levels to increase access.	*“It’ll get grandma or my mom to do it, if it is in Spanish”* (*Latina, Participant, Female;* Ridley-Merriweather 2022 p.6)	Aranda 2023, Frierson 2019, Niranjan 2019, Regnante 2020, Ridley-Merriweather 2022, Rivers 2019, Schatz 2023, TaPark 2023
**Patient Navigation**	Assists participants in better navigating and understanding clinical research.	*“Someone who would know about the clinical trial because I think that would be a qualification in order to be a navigator. That’s my primary goal cause I would want to have someone with some type of information that would have a beginning and middle and ending that’s been or that’s walking through the different procedures that the clinical trial and what to expect and not to expect” (African American, Participant;* Hernandez, 2021, p.6).	Dance 2021, Hartley-Brown 2024, Hernandez 2021, Joseph 2009, Niranjan 2021, Schatz 2023, Vickers 2023
**Patient-Provider Relationships**	Uses providers’ expertise and rapport for outreach and communication through leveraging patient-provider relationships.	*“…I think that was the decision I made: either I’m gonna trust her or I’m not. If I’m not gonna trust her, I shouldn’t get the treatment. So, I made up my mind from the very beginning that I trusted my doctor” (African American, Participant, Male, 73;* Dance 2021, p.1862).	An 2023, Dance 2021, Fink 2023, Haynes-Maslow 2014, Joseph 2009, Legor 2023, Mesa 2023, Regnante 2020, Stockdill 2023
**Social Networks**	Incorporates and targets peer groups, family, friends or other social networks.	*“Latinas are more wanting to think in terms of family” (Latina, Participant, Female;* Ridley-Merriweather 2022, p.7). *“My family or really close friends, if it helps them then yeah I’ll consider it” (Asian, Participant, Female;* Ridley-Merriweather 2022, p.7).	Alvarado 2023, Fink 2023, Hernandez 2021, Portacolone 2020, Regnante 2020, Ridley- Merriweather 2022, TaPark 2023
**Systems Approach**	Utilizes systems thinking and structural approaches such as creating databases that collect/monitor race and ethnicity data over time; and ensuring inclusive clinical research criteria, procedures and policies.	*“Oh I think we can, we definitely absolutely can and we do measure how many minorities we put on studies but I don’t think we’re actively seeking to increase that number” (Research Staff;* Niranjan 2021, p.669).	Aranda 2023, Crabbe 2023, Lincoln 2021, Niranjan 2021, Regnante 2020, TaPark 2023
**Technology**	Uses technology including social media, videos, photos, graphics, media, information systems, and other methods for outreach and to increase education and communication.	*“… that part where they {in the video} talked about Tuskegee … It’s nice they touched on that and acknowledged it, especially for African American people....” (African American, Participant, Female;* Rivers 2019, p.7).	An 2023, Aranda 2023, Hartley-Brown 2024, Portacolone 2020, Rivers 2019, Robinson 2020, Stockdill 2023, Vickers 2023
**Training**	Trains clinical and research staff about inclusive research frameworks.	*“I think that that {training} would be useful for anybody who’s dealing with clinical trials and accruing patients, so that would be coordinators, nurses, physicians, you know. I think it would because some of the concerns, as challenging as I am a very pasty white Irish person I don’t want someone to feel that I am either talking down to them or that I don’t understand what their concerns are and be able to talk to a patient in a way that doesn’t come off as condescending or too authoritative, dictator kind of, you need to do this, and it’s very hard for us to walk in someone else’s shoes, and so for additional training if it makes me a better communicator and help to build trust with the patient I am all for anything that does that” (White, Research Staff; s*Niranjan 2019, p.30).	Hartley-Brown 2024, Legor 2023, Lincoln 2021, Niranjan 2019, Schatz 2023

* Indicates identity characteristics are omitted as these identifiers were not shared in original paper

{} Indicates our study team inserted additional text for clarity.

This section summarizes the high-level interpretation and summary of themes derived from the inductive coding. Themes critical for representative clinical research among groups less represented in clinical research included empowerment, sustainability, transparency, and trust. Empowerment explores how education, communication, or other tools seek to empower participants to be involved in clinical research. This includes how participants are empowered to make decisions to engage in clinical research based upon informed decisions, patient navigation, advocating for themselves, and other tools seeking to bolster local capacity through partnerships, collaboration, and community engagement. Within this theme, participants exhibit a sense of empowerment through demonstrating autonomy in making informed decisions about engaging in clinical research. Trust highlights the importance of clinical research staff developing trust with participants or the community. This can include respecting community boundaries, culture, and norms; demonstrating respect through connections, communication, and consistent and repeated contact/positive interactions; using community-based spaces to foster trust for participants; and building trust through enhancing rapport. Transparency demonstrates the need for clear communication about clinical research including benefits and risks, health information, and additional information supporting informed decision-making. Sustainability describes long-term investment into fostering relationships with the community, building sustainable infrastructure such as a systems approach, developing procedures such as databases and registries, and implementing policy approaches to support enhanced engagement among these populations in clinical research.

**Fig 2:**
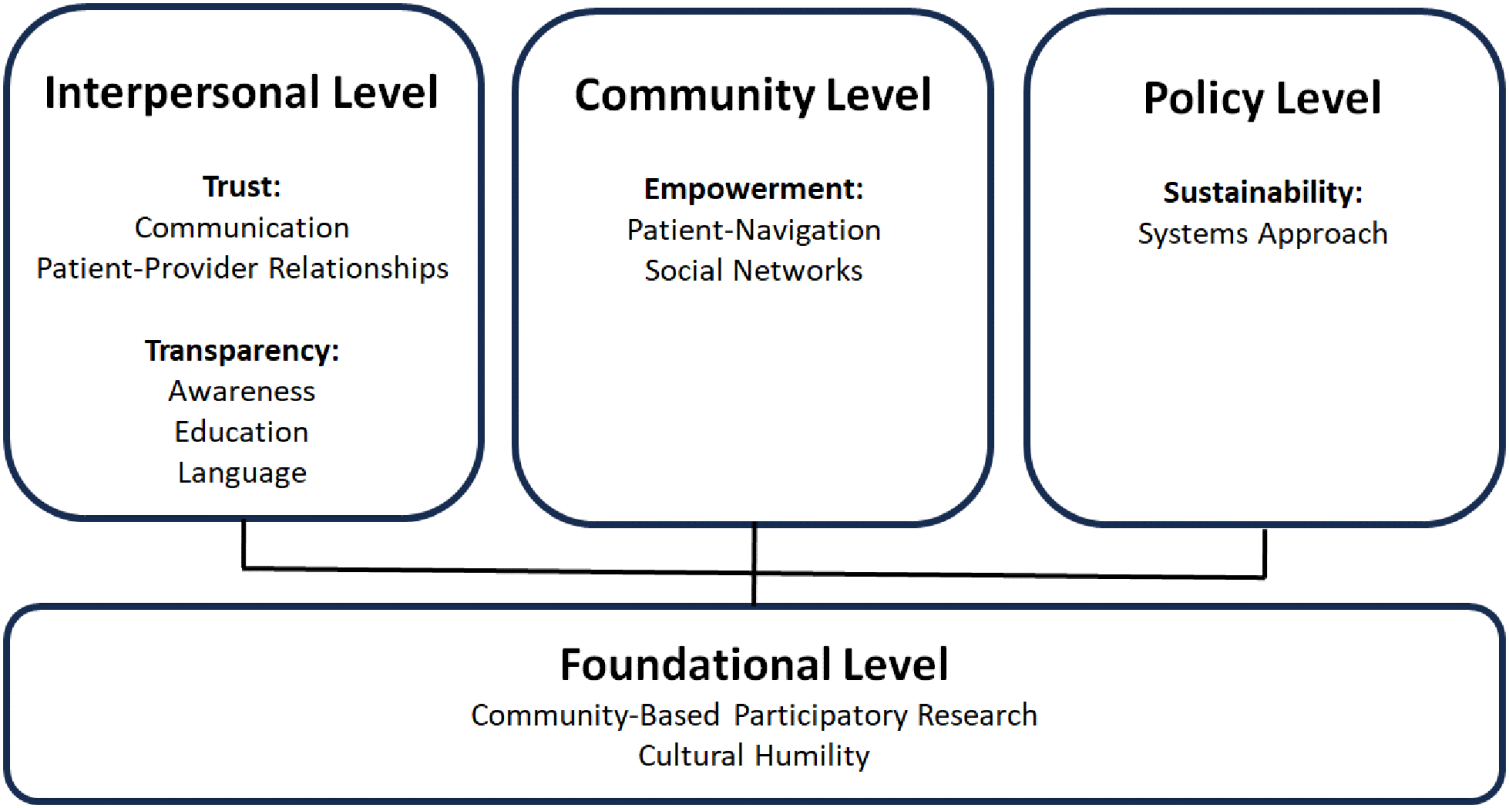
Conceptual model of strategies for participation of groups less represented in clinical research. Interpersonal, community, and policy levels adapted from the socioecological model. Bold text = themes from analysis. Training is embedded primarily within the foundation of Cultural Humility and Interpersonal Level of Trust within Patient-Provider relationships. Technology can be applied at all levels, but its use is evolving rapidly and needs to be considered carefully within the context of Cultural Humility and CBPR.

Fig 2 depicts our conceptual model of recruitment and retention strategies for groups less represented in clinical research, that is an assessment of these aforementioned themes and strategies. This model explores both the strategies reflected in our deductive coding, along with themes derived from our inductive coding. With roles in our meta-synthesis systematic review spanning from research participants to medical center and community leaders, our analysis pointed to the critical need for multi-level approaches. Therefore, our model adapts the socioecological model levels of interpersonal, community, and policy [54]. The interpersonal level demonstrates the themes of trust as well as transparency, with strategies such as communication, patient-provider relationships, awareness, education, and language being most prominent. The community level primarily exhibits the theme of empowerment, with strategies such as patient-navigation and social networks playing primary roles. And, the policy level presents the theme of sustainability, with strategies of a systems approach to reduce research engagement barriers being most pronounced. At the foundation, and essential across all levels, are cultural humility and Community-Based Participatory Research (CBPR).

## Discussion

This is one of the first systematic reviews to qualitatively assess strategies that seek to increase clinical research participation among less represented groups, across the leading causes of morbidity and mortality in the U.S. The initial review encompassed literature from 2009–2020. As the study progressed, additional updates were performed in 2022 and 2024 to include more recent literature. The review includes perspectives spanning the clinical research ecosystem, with emphasis primarily on recruitment, and cancer. Strategies identified along with themes derived from high level interpretation emphasize the need for efforts at multiple levels concentrating on CBPR and cultural humility. This meta-synthesis systematic review: (1) provides qualitative data about evidence-based strategies to recruit and retain groups less represented in clinical research; (2) reveals remaining opportunities and gaps; and (3) details actions for creating a culture of equitable clinical research.

In our review, the strategy that was most frequently used to increase the participation of these populations in clinical research was CBPR; nearly 63% of the studies in our review utilized this strategy (S3 Table). This included direct engagement with the community (58.8%), partnership with community leaders (52.9%), engaging with community-based organizations (17.6%), and receiving feedback from community leaders (17.6%). This highlights the importance of building and maintaining a relationship with the community, from the initiation of study design through the completion of the study. Cultural humility, which was also identified as a strategy, is needed to initiate and maintain a relationship with the community. Cultural humility requires the research team to remove their own assumptions and become the student that respectfully learns how the culture and concerns of potential participants impacts their health behaviors. This approach allows the study team to engage a community, identify potential concerns, design, and implement a study that is culturally sensitive and tailored for those participants. In practice, this may include: (1) providing tailored information, material, and consent forms in the relevant language; (2) using relevant and respectful imagery in study material; or (3) considering practices and beliefs in study design. According to our conceptual model, cultural humility partnered with CBPR is foundational to the recruitment and retention of these populations at the interpersonal, community, and policy level.

Nearly half of the studies in our review noted that clear, consistent communication and education enhanced recruitment and retention of these populations (S3 Table). Effectively communicating and educating participants on study information (i.e., the purpose, disease, study requirements, appointment schedule, risks, benefits, study results) in a manner that is patient, non-condescending, and relatable, was considered an effective technique. Utilizing storytelling techniques to communicate the benefits and risks of the study, was also shown to be an effective strategy. One participant expressed their desire to know more about the study, beyond what is included in the informed consent, emphasizing the utility of clear communication (Table 2). Educating the participant network using programs, flyers, posters, or pamphlets was named as useful strategies. Other strategies that were identified through this review include: (1) supportive patient-provider relationship, (2) use of relevant languages, (3) use of technology like social media and visuals, (4) use of patient navigation, (5) use of social networks, (6) use of systems approach, and (7) training of clinical research staff. While this review focused on studies performed in the U.S., these strategies are applicable to clinical research studies that are performed outside of the U.S., that aim to recruit study participants. There are recommendations and studies from groups outside the U.S. that highlight the use of the strategies identified in this review, for clinical research in countries beyond the U.S. [55,56].

These findings align with our unpublished qualitative analysis, that examined NIH-funded clinical studies to identify strategies that improved recruitment and retention of populations in clinical research. This study surveyed NIH institutes to identify clinical study exemplars that had achieved participation of less represented populations in their study. We collected data from 19 of NIH’s 27 institutes, resulting in the identification of 123 exemplars. Our analysis revealed that the study sites intentionally built and sustained relationships with the community of interest. These sites also had study teams that were representative of the patient population, that contributed to the successful recruitment and retention of these study participants. The exemplar studies collected and monitored the demographic data of study participants. They frequently examined these numbers during the study and adjusted their strategies to ensure that the study enrollment matched the incidence rates of the condition being studied. Lastly, these studies fostered transparency, accountability, and frequent communication between the sponsor and research team as well as between the research team and the study participants. This aligns with the themes identified in our meta-synthesis that are critical for representative clinical research: empowerment, sustainability, transparency, respect, and trust.

This review proceeded concurrently with the recent NASEM report entitled *Improving Representation in Clinical Trials and Research: Building Research Equity for Women and Underrepresented Groups* [12]. The scope of the NASEM report extends far beyond our review. However, we have achieved a primary objective set forth in *Chapter 5: Facilitators of Successful Inclusion in Clinical Research* and provide evidence-based facilitators of appropriate representation in clinical research (Table 2). Our work complements the NASEM report, which focused on research teams, by centering the voices of persons involved across the continuum of clinical research, i.e., participants, caregivers, clinicians, and community leaders (S2 Table).

Our review clarifies opportunities for future scholarship. Previous work has focused on obstacles to participation and how to overcome these challenges in clinical research in general. However, there is a paucity of literature assessing strategies across communities and diseases. Although this review was unable to represent all groups and conditions, this does not imply there is no work being done; rather, the work may be at stages that did not fit the current criteria (e.g., studies identifying obstacles or collecting community feedback about the strategies being tested). The work presented here does highlight the opportunity to assess strategies across populations and diseases as a potential indicator for directly replicating them in a new setting or adapting before implementation. For example, CBPR strengthened by cultural humility [57] were decisively emphasized and, hence, were considered to be foundational (Fig 2). This conclusion is consistent with previous findings related to enhancing engagement of in clinical research [58–61] and is reflected in the recent NASEM report. In centering the community norms, values, voices, and collaborating with the community, CBPR and cultural humility permeate our conceptual model at all levels. One way that cultural humility acts in-service to CBPR is reflected in the evolving landscape of CBPR which recognizes the need for a continuing focus on balancing power in partnerships (e.g., by sharing decision making authority with the community [62]).

There are populations whose perspectives are vital but were not reflected in the studies that are included in this review. Notably absent from the studies included in our review are the perspectives from American Indian/Alaska Native (AI/AN) populations. Qualitative data from AI/AN groups will clarify tailored and adapted strategies unique to these groups. The perspective of young participants (i.e., under the age of 18) was also not represented in the literature included in our review. Given NIH’s Inclusion Across the Lifespan Policy that expanded the inclusion of children in clinical research, having that data would provide guidance on how to design pediatric clinical research studies. Hearing from young persons from less represented groups also has implications for diseases originally considered for this review that are prevalent in youth (e.g., asthma [63]). Also, how each generation relates to the clinical research ecosystem may be unique due to the historical policy, context, and technological advances and therefore will require customized interactions [64]. Finally, the perspective from industry was also absent from the literature in our review. Understanding the perspective of industry is critical, since a vast majority of clinical trials are performed by companies [65].

Lastly, a limitation of our review is that it consisted of studies reporting recruitment strategies but did not include studies with retention strategies. Future work comparing such results is critical when considering Phase 3 clinical trials for drugs and devices typically last 1–4 years [66]. Given the time commitment, size, and cost associated with Phase 3 clinical trials, the theme of sustainability is likely to play a pivotal role in strategies for retention [67–69].

Actions to create representative clinical research proceeded from our thematic analysis and conceptual model. The actions described here should be initiated, rigorously evaluated, and accelerated by establishing and sustaining a community of practice focused on recruitment and retention of less represented groups in clinical research. The community of practice must include representatives from across populations, sectors (government, academia, industry, non-profit, publishing, philanthropic institutions, etc.), and roles in the clinical research ecosystem. The community of practice should be accessible for timely consultation to researchers.

There is a need to engage in thoughtful deliberations about the time, personnel, and funding levels necessary to create a culture of representative clinical research and promote population healthy, via CBPR strengthened by cultural humility. This sentiment was also clearly stated in the NASEM report [12]. From such deliberation, innovative partnership structures spanning sectors may be required (e.g., work analogous to the Framingham Heart Study) [70].

Given the magnitude of health disparities, the future generation of researchers (including undergraduate and graduate students) should receive earlier course curriculum on the importance of representative clinical research. Topics, embedded in science-technology-engineering-mathematics-medicine (STEMM) based pedagogies might include, but are not limited to: 1) acknowledging multi-faceted obstacles to representative clinical research; 2) the facilitators that improve participation of populations in clinical research and how to implement them; 3) understanding how not having representative clinical studies prevents the development of health care innovations [71]; and, 4) understanding that improving clinical research overall could advance but will achieve population health.

It is critical to consolidate and actively disseminate resources about recruitment and retention strategies through recurring educational sessions or creating clinical research offices that provide consultation for researchers. This would allow researchers to promptly replicate and/or adapt strategies for their clinical research ecosystem. To ensure accountability, standards for and reporting of representative clinical research should be considered. Example standards might include, but are not limited to defining appropriate reference numbers for appropriate representation, e.g., population versus disease demographics; intentionally collecting and reporting qualitative feedback on strategies employed; and disaggregating findings by demographic data and role in clinical research ecosystem, etc. To disseminate actionable strategies, partners could develop curricula, hold workshops, and convene summits to share best practices and methods.

Strategies to recruit and retain populations in clinical research must prioritize the needs of the community and operationalize a sustainable multi-level approach where both cultural humility and community-based participatory research are foundational for effective implementation. To create representative clinical research, a re-energized focus needs to include earlier career training to make implementation of strategies intuitive for the next generations of researchers; establishment and monitoring of standards and reporting; and thoughtful deliberation about the time, personnel, and funding levels necessary to advance population health.

## Supporting information

S1 Appendix Prisma 2020 Checklist. Diagram illustrates the selection procedure for studies included in the systematic review. It follows the PRISMA (Preferred Reporting Items for Systematic Reviews and Meta-Analyses) guidelines.
(DOCX)

S1 Table Inclusion Criteria. Summary of inclusion criteria that was used for this systematic review.
(DOCX)

S2 Appendix Search Strategy. For our search strategy, the search terms included: underrepresented, minority, racial and ethnic groups, clinical research, and disparities. The search limits include the following: publication year of 2009–2024, language of English and Spanish, geography of United States only, and species of human studies not inclusive of in vitro studies. Search terms are included. No search filters were operationalized.
(DOCX)

S3 Appendix All Studies Identified in Initial Search. A comprehensive list of all literature (4,756 studies) that were identified in the initial search. The literature screened out during the title and abstract screening (3842 studies), excluded during the full text review (887 studies), and included in the systematic review (27 studies) are desginated accordingly.
(XLSX)

S4 Appendix Quality Assessment. Quality assessment for each study included in the review. The table list all the studies that were included in the systematic review and the questions that were asked to assess whether the study partially met or fully met all criteria.
(DOCX)

S5 Appendix All Extracted Data from Included Studies. Data extracted from the primary research sources that were included in this systematic review. This details study characteristics including race/ethnicity, condition of interest, sex where noted, role in clinical research (e.g., participant, clinician (i.e., medical or research staff), community leader, etc.), geography of study (urban or rural), the name of the data extractors, date of data extraction, and the confirmation that the study was eligible for inclusion.
(DOCX)

S2 Table Characteristics of included studies for meta-synthesis of evidence-based strategies (N = 27). Inclusion criteria (study purpose – recruitment or retention, race and ethnicity, condition) are separated from other data extracted during the review. If an entry is missing, then the characteristic is unreported and/or not disaggregated. F = female, M = Male. SD = standard deviation. PI = principal investigator. Study partners = typically a family member or close friend. CBO Admin = community-based organization administrator. * Data in Niranjan references appear to be derived from the same Sources and therefore counted only once when totaling individuals reflected in this meta-synthesis. Quality assessment was conducted, where studies either met all or partially met criteria per on Critical Appraisal Skills Programme. Studies that partially met criteria remained included given their relevancy. The most common reason for partially met criteria was related to the sampling method.
(DOCX)

S3 Table Summary of recruitment strategies for groups less represented in clinical research. American Indian/Alaska Native are not listed because no studies included these populations. Niranjan 2019 and Fink 2023, focused on both recruitment and retention, were combined as recruitment for this table. Ridley-Merriweather 2022 focused on disaggregated strategies for both Asian and Latinos and TaPark 2023 focused on disaggregated strategies for both Asian and Native Hawaiian/Pacific Islander. There are 12 strategies, each listed in bold as headers. For each strategy, there are corresponding subcategories. In total, 27 studies were included in the analysis. The subcategories can be up to the maximum total for each strategy header. The subcategories may be counted multiple times for each theme.
(DOCX)
