# Supplementary material for: Recruitment and retention strategies for improving representation in clinical research: A meta-synthesis
Source: PLoS One. 2025 Jun 23;20(6):e0322796. doi: 10.1371/journal.pone.0322796 (PMC12184919; doi:10.1371/journal.pone.0322796)
Supplement: S1 Table — Summary of inclusion criteria that was used for this systematic review. (DOCX) [file pone.0322796.s002.docx]

**S1 Table. Inclusion criteria.**

| **Recruitment and retention** |
| --- |
| **Ethnic and racial minorities**: Race or ethnic minorities in the U.S. and NIH designated health disparity populations  (American Indian or Alaska Native, Asian, Black/African American, Native Hawaiian or Other Pacific Islander, and ethnic minorities as Hispanic/Latino) |
| **Leading causes of morbidity and mortality in the United States:** Heart disease, cancer, chronic respiratory diseases, stroke, diabetes, Alzheimer’s disease, pneumonia and influenza, and kidney disease |
| **All ages** |
| **Clinical research**  Research with human subjects that is:  1. Patient-oriented research. Research conducted with human subjects (or on material of human origin such as tissues, specimens, and cognitive phenomena) for which an investigator (or colleague) directly interacts with human subjects. Excluded from this definition are in vitro studies that utilize human tissues that cannot be linked to a living individual. It includes: (a) mechanisms of human disease, (b), therapeutic interventions, (c) clinical trials, or (d) development of new technologies.  2. Epidemiological and behavioral studies.  3. Outcomes research and health services research  Studies falling under 45 CFR 46.101(b) (4) (Exemption 4) are not considered clinical research by this definition. |
| **Years**: 2009-2024 |
| **Written in English or Spanish** |
| **Conducted in the United States** |
