# Supplementary material for: Recruitment and retention strategies for improving representation in clinical research: A meta-synthesis
Source: PLoS One. 2025 Jun 23;20(6):e0322796. doi: 10.1371/journal.pone.0322796 (PMC12184919; doi:10.1371/journal.pone.0322796)
Supplement: S2 Appendix — For our search strategy, the search terms included: underrepresented, minority, racial and ethnic groups, clinical research, and disparities. The search limits include the following: publication year of 2009–2024, language of English and Spanish, geography of United States only, and species of human studies not inclusive of in vitro studies. Search terms are included. No search filters were operationalized. (DOCX) [file pone.0322796.s003.docx]

S2 Appendix. Search Strategy.

**Citation/Title of Review:** Evidence-Based Recruitment and Retention Strategies for Racial and Ethnic Groups Underrepresented in Clinical Research: A Meta-Synthesis

**Database:** PubMed/MEDLINE

**Platform:** US National Library of Medicine

**Date of Original Search:** April 29, 2021

**Date of Search Update 1**: May 18, 2022

**Date of Search Update 2:** Jan 25, 2024

**Original Search Limits:** MEDLINE subset; Language: English, Spanish; Publication date: 1/1/2009 – 12/31/2020

**Search Update 1 Limits:** MEDLINE subset; Language: English, Spanish; Publication date: 1/1/2021 – 05/18/2022

**Search Update 2 Limits:** MEDLINE subset; Language: English, Spanish; Publication date: 1/1/2022 – 01/25/2024

**Notes:** The PubMed search strategy is the same for the **original search** and **search update 1** – only the date limits were changed. Keywords and corresponding PubMed MeSH thesaurus terms were used to search for each concept. Each search strategy was run in the order specified and then applied the limits as indicated to exclude animal studies and specific publication types as specified in the exclusion criteria.

| **PubMed Search Strategy – Original Search and Search Update 1** |
| --- |
| #1) (disparit*[tiab] OR underrepresent*[tiab] OR "under represent*"[tiab] OR minority[tiab] OR minorities[tiab] OR "special population*"[tiab] OR “ethnic group*”[tiab] OR “racial group*”[tiab] OR "African American*"[tiab] OR black[tiab] OR blacks[tiab] OR Hispanic*[tiab] OR Latino*[tiab] OR Latina*[tiab] OR "American Indian*"[tiab] OR "native American*"[tiab] OR "Alaska native*"[tiab] OR “Asian American*”[tiab] OR "native Hawaiian*"[tiab] OR "pacific islander*"[tiab] OR guamanian*[tiab] OR chamorro*[tiab] OR samoan*[tiab] OR "Mexican American*"[tiab] OR chicano*[tiab] OR chicana*[tiab] OR "Puerto Rican*"[tiab] OR "Cuban american*"[tiab] OR "Minority Groups"[Mesh] OR "African Americans"[Mesh] OR Ethnic Groups[Mesh] OR "Asian Americans"[Mesh] OR Hispanic Americans[Mesh] OR "Mexican Americans"[Mesh] OR "Alaskan Natives"[Mesh] OR "Indians, North American"[Mesh] OR American Natives[Mesh] OR Continental Population Groups[majr])  #2 ("clinical research"[tiab] OR "clinical study"[tiab] OR "clinical studies"[tiab] OR "clinical trial*"[tiab] OR “controlled trial*”[tiab] OR "Clinical Trials as Topic"[Mesh] OR "Clinical Studies as Topic"[Mesh] OR "Randomized Controlled Trials as Topic"[Mesh] OR "Controlled Clinical Trials as Topic"[Mesh]) AND (underenrol*[tiab] OR “under-enroll*”[tiab] OR participat*[tiab] OR participation[tiab] OR recruit[tiab] OR recruits[tiab] OR recruited[tiab] OR recruiting[tiab] OR recruitment*[tiab] OR retain*[tiab] OR retention[tiab] OR enrol*[tiab] OR enroll*[tiab] OR engagement*[tiab] OR engage*[tiab] OR engaging[tiab] OR "patient selection"[tiab] OR "selecting patient*"[tiab] OR barrier*[ti] OR facilitate*[ti] OR "Patient Participation"[mesh] OR "patient selection"[mesh])    #3("heart disease*"[tiab] OR "Heart Diseases"[Majr] OR cancer*[tiab] OR neoplasm*[tiab] OR carcinoma*[tiab] OR "Neoplasms"[Majr] OR stroke[tiab] OR strokes[tiab] OR "Cerebrovascular Accident*"[tiab] OR "Stroke"[Majr] OR diabetes[tiab] OR "Diabetes Mellitus"[Majr] OR "Alzheimer disease*"[tiab] OR "alzheimers disease"[tiab] OR "Alzheimer dementia*"[tiab] OR "alzheimers dementia*"[tiab] OR "Alzheimer Disease"[Majr] OR pneumonia*[tiab] OR "Pneumonia"[Majr] OR influenza*[tiab] OR "Influenza, Human"[Majr] OR "kidney disease*"[tiab] OR "Kidney Diseases"[Majr] OR "chronic obstructive pulmonary disease*"[tiab] OR COPD[tiab] OR "Pulmonary Disease, Chronic Obstructive"[Majr] OR "pulmonary hypertension"[tiab] OR "Hypertension, Pulmonary"[Majr] OR asthma[tiab] OR "Asthma"[Majr])  #4) ((alabama[tiab] or alaska[tiab] or arizona[tiab] or arkansas[tiab] or california[tiab] or colorado[tiab] or connecticut[tiab] or delaware[tiab] or florida[tiab] or georgia[tiab] or hawaii[tiab] or idaho[tiab] or illinois[tiab] or indiana[tiab] or iowa[tiab] or kansas[tiab] or kentucky[tiab] or louisiana[tiab] or maine[tiab] or maryland[tiab] or massachusetts[tiab] or michigan[tiab] or minnesota[tiab] or mississippi[tiab] or missouri[tiab] or montana[tiab] or nebraska[tiab] or nevada[tiab] or "new hampshire"[tiab] or "new jersey"[tiab] or "new mexico"[tiab] or "new york"[tiab] or "north carolina"[tiab] or "north dakota"[tiab] or ohio[tiab] or oklahoma[tiab] or oregon[tiab] or pennsylvania[tiab] or "rhode island"[tiab] or "south carolina"[tiab] or "south dakota"[tiab] or tennessee[tiab] or texas[tiab] or utah[tiab] or vermont[tiab] or virginia[tiab] or washington[tiab] or "west virginia"[tiab] or wisconsin[tiab] or wyoming[tiab] or usa[tiab] or "united states"[tiab] or us[tiab] or u.s.[tiab] OR u.s.a.[tiab] OR American*[tiab] OR "New York City"[tiab] OR "Los Angeles"[tiab] OR Houston[tiab] OR Dallas[tiab] OR Miami[tiab] OR Chicago[tiab] OR Dallas[tiab] OR Detroit[tiab] OR Seattle[tiab] OR "San Francisco"[tiab] OR Sacramento[tiab] OR "San Diego"[tiab] OR Boston[tiab] OR "Washington DC"[tiab] OR Baltimore[tiab] OR Pittsburgh[tiab] OR Philadelphia[tiab] OR Denver[tiab] OR Phoenix[tiab] OR "New Orleans"[tiab] OR "American Samoa"[tiab] OR "Northern Mariana Islands"[tiab] or guam[tiab] or "puerto rico"[tiab] or "virgin islands"[tiab] or alabama[mesh] or alaska[mesh] or arizona[mesh] or arkansas[mesh] or california[mesh] or colorado[mesh] or connecticut[mesh] or delaware[mesh] or florida[mesh] or georgia[mesh] or hawaii[mesh] or idaho[mesh] or illinois[mesh] or indiana[mesh] or iowa[mesh] or kansas[mesh] or kentucky[mesh] or louisiana[mesh] or maine[mesh] or maryland[mesh] or massachusetts[mesh] or michigan[mesh] or minnesota[mesh] or mississippi[mesh] or missouri[mesh] or montana[mesh] or nebraska[mesh] or nevada[mesh] or "new hampshire"[mesh] or "new jersey"[mesh] or "new mexico"[mesh] or "new york"[mesh] or "north carolina"[mesh] or "north dakota"[mesh] or ohio[mesh] or oklahoma[mesh] or oregon[mesh] or pennsylvania[mesh] or "rhode island"[mesh] or "south carolina"[mesh] or "south dakota"[mesh] or tennessee[mesh] or texas[mesh] or utah[mesh] or vermont[mesh] or virginia[mesh] or washington[mesh] or "west virginia"[mesh] or wisconsin[mesh] or wyoming[mesh] or "united states"[mesh] or "new york city"[mesh] or "district of columbia"[mesh] or "philadelphia"[mesh] or "baltimore"[mesh] or boston[mesh] or chicago[mesh] or "los angeles"[mesh] or "new orleans"[mesh] or "san francisco"[mesh] or "american samoa"[mesh] or guam[mesh] or "puerto rico"[mesh] or "united states virgin islands"[mesh] or alabama[ad] or alaska[ad] or arizona[ad] or arkansas[ad] or california[ad] or colorado[ad] or connecticut[ad] or delaware[ad] or florida[ad] or georgia[ad] or hawaii[ad] or idaho[ad] or illinois[ad] or indiana[ad] or iowa[ad] or kansas[ad] or kentucky[ad] or louisiana[ad] or maine[ad] or maryland[ad] or massachusetts[ad] or michigan[ad] or minnesota[ad] or mississippi[ad] or missouri[ad] or montana[ad] or nebraska[ad] or nevada[ad] or "new hampshire"[ad] or "new jersey"[ad] or "new mexico"[ad] or "new york"[ad] or "north carolina"[ad] or "north dakota"[ad] or ohio[ad] or oklahoma[ad] or oregon[ad] or pennsylvania[ad] or "rhode island"[ad] or "south carolina"[ad] or "south dakota"[ad] or tennessee[ad] or texas[ad] or utah[ad] or vermont[ad] or virginia[ad] or washington[ad] or "west virginia"[ad] or wisconsin[ad] or wyoming[ad] or guam[ad] or "puerto rico"[ad] or “american samoa”[ad] or “northern mariana islands”[ad] or "united states virgin islands"[ad] or "united states"[ad] or usa[ad])  #5) #1 AND #2 AND #3 AND #4  #6 ) #5 NOT (mice[tiab] OR mouse[tiab] OR rat[tiab] OR rats[tiab] OR rodent*[tiab] OR dog[tiab] OR dogs[tiab] OR pig[tiab] OR pigs[tiab] OR piglet*[tiab] OR swine[tiab] OR porcine*[tiab] OR animal*[tiab]) NOT ("Animals"[Mesh] NOT ("Animals"[Mesh] AND "Humans"[Mesh])) NOT (letter[ptyp] OR editorial[ptyp] OR comment[ptyp] OR news[ptyp] OR editorial[tiab] OR commentary[tiab] OR "retracted publication"[ptyp] OR "retraction of publication"[ptyp] OR "retraction of publication"[tiab] OR "retraction notice"[ti] OR "retracted publication"[tiab] OR "Published Erratum"[Publication Type] OR errata[tiab] OR erratum[tiab] OR corrigenda[tiab] OR corrigendum[tiab] OR protocol[ti] OR protocols[ti] OR "case report*"[tiab] OR "Case Reports" [Publication Type] OR "Congress"[Publication Type] OR "Consensus Development Conference"[Publication Type] OR editorial[tiab] OR commentary[tiab] OR “conference abstract*”[tiab] OR “conference proceeding*”[tiab]) AND (English[lang] OR Spanish[lang])  Date Filters: Original Search : 1/1/2009 – 12/31/2020 Date Filters: Search Update 1 - 1/1/2021 – 05/18/2022 |

**Database:** PubMed/MEDLINE

**Platform:** US National Library of Medicine

**Search Update 2 Limits:** MEDLINE subset; Language: English, Spanish; Publication date: 1/1/2022 – 01/25/2024

**Note**:

- The search strategy for **search update 2** is listed below.
- The strategy was modified slightly to reflect updated PubMed MeSH thesaurus terms (highlighted in yellow below) The remaining search strategy terms stayed the same.
- Date limits were changed to reflect the second search update.

| **Search Strategy – Search Update 2 (with updated PubMed Mesh Terms)** |
| --- |
| #1) **(disparit*[tiab] OR underrepresent*[tiab] OR "under represent*"[tiab] OR minority[tiab] OR minorities[tiab] OR "special population*"[tiab] OR "ethnic group*"[tiab] OR "racial group*"[tiab] OR "African American*"[tiab] OR black[tiab] OR blacks[tiab] OR Hispanic*[tiab] OR Latino*[tiab] OR Latina*[tiab] OR "American Indian*"[tiab] OR "native American*"[tiab] OR "Alaska native*"[tiab] OR "Asian American*"[tiab] OR "native Hawaiian*"[tiab] OR "pacific islander*"[tiab] OR guamanian*[tiab] OR chamorro*[tiab] OR samoan*[tiab] OR "Mexican American*"[tiab] OR chicano*[tiab] OR chicana*[tiab] OR "Puerto Rican*"[tiab] OR "Cuban american*"[tiab] OR "Minority Groups"[Mesh] OR "Black or African American"[Mesh] OR "Asian"[Mesh] OR "Hispanic or Latino"[Mesh] OR ethnicity[Mesh] OR "Mexican Americans"[Mesh] OR "Alaska Natives"[Mesh] OR "Indians, North American"[Mesh] OR "American Indian or Alaska Native"[Mesh] OR Continental Population Groups[majr] OR Population Groups, US[Mesh])**  #2 ("clinical research"[tiab] OR "clinical study"[tiab] OR "clinical studies"[tiab] OR "clinical trial*"[tiab] OR “controlled trial*”[tiab] OR "Clinical Trials as Topic"[Mesh] OR "Clinical Studies as Topic"[Mesh] OR "Randomized Controlled Trials as Topic"[Mesh] OR "Controlled Clinical Trials as Topic"[Mesh]) AND (underenrol*[tiab] OR “under-enroll*”[tiab] OR participat*[tiab] OR participation[tiab] OR recruit[tiab] OR recruits[tiab] OR recruited[tiab] OR recruiting[tiab] OR recruitment*[tiab] OR retain*[tiab] OR retention[tiab] OR enrol*[tiab] OR enroll*[tiab] OR engagement*[tiab] OR engage*[tiab] OR engaging[tiab] OR "patient selection"[tiab] OR "selecting patient*"[tiab] OR barrier*[ti] OR facilitate*[ti] OR "Patient Participation"[mesh] OR "patient selection"[mesh])    #3("heart disease*"[tiab] OR "Heart Diseases"[Majr] OR cancer*[tiab] OR neoplasm*[tiab] OR carcinoma*[tiab] OR "Neoplasms"[Majr] OR stroke[tiab] OR strokes[tiab] OR "Cerebrovascular Accident*"[tiab] OR "Stroke"[Majr] OR diabetes[tiab] OR "Diabetes Mellitus"[Majr] OR "Alzheimer disease*"[tiab] OR "alzheimers disease"[tiab] OR "Alzheimer dementia*"[tiab] OR "alzheimers dementia*"[tiab] OR "Alzheimer Disease"[Majr] OR pneumonia*[tiab] OR "Pneumonia"[Majr] OR influenza*[tiab] OR "Influenza, Human"[Majr] OR "kidney disease*"[tiab] OR "Kidney Diseases"[Majr] OR "chronic obstructive pulmonary disease*"[tiab] OR COPD[tiab] OR "Pulmonary Disease, Chronic Obstructive"[Majr] OR "pulmonary hypertension"[tiab] OR "Hypertension, Pulmonary"[Majr] OR asthma[tiab] OR "Asthma"[Majr])  #4) ((alabama[tiab] or alaska[tiab] or arizona[tiab] or arkansas[tiab] or california[tiab] or colorado[tiab] or connecticut[tiab] or delaware[tiab] or florida[tiab] or georgia[tiab] or hawaii[tiab] or idaho[tiab] or illinois[tiab] or indiana[tiab] or iowa[tiab] or kansas[tiab] or kentucky[tiab] or louisiana[tiab] or maine[tiab] or maryland[tiab] or massachusetts[tiab] or michigan[tiab] or minnesota[tiab] or mississippi[tiab] or missouri[tiab] or montana[tiab] or nebraska[tiab] or nevada[tiab] or "new hampshire"[tiab] or "new jersey"[tiab] or "new mexico"[tiab] or "new york"[tiab] or "north carolina"[tiab] or "north dakota"[tiab] or ohio[tiab] or oklahoma[tiab] or oregon[tiab] or pennsylvania[tiab] or "rhode island"[tiab] or "south carolina"[tiab] or "south dakota"[tiab] or tennessee[tiab] or texas[tiab] or utah[tiab] or vermont[tiab] or virginia[tiab] or washington[tiab] or "west virginia"[tiab] or wisconsin[tiab] or wyoming[tiab] or usa[tiab] or "united states"[tiab] or us[tiab] or u.s.[tiab] OR u.s.a.[tiab] OR American*[tiab] OR "New York City"[tiab] OR "Los Angeles"[tiab] OR Houston[tiab] OR Dallas[tiab] OR Miami[tiab] OR Chicago[tiab] OR Dallas[tiab] OR Detroit[tiab] OR Seattle[tiab] OR "San Francisco"[tiab] OR Sacramento[tiab] OR "San Diego"[tiab] OR Boston[tiab] OR "Washington DC"[tiab] OR Baltimore[tiab] OR Pittsburgh[tiab] OR Philadelphia[tiab] OR Denver[tiab] OR Phoenix[tiab] OR "New Orleans"[tiab] OR "American Samoa"[tiab] OR "Northern Mariana Islands"[tiab] or guam[tiab] or "puerto rico"[tiab] or "virgin islands"[tiab] or alabama[mesh] or alaska[mesh] or arizona[mesh] or arkansas[mesh] or california[mesh] or colorado[mesh] or connecticut[mesh] or delaware[mesh] or florida[mesh] or georgia[mesh] or hawaii[mesh] or idaho[mesh] or illinois[mesh] or indiana[mesh] or iowa[mesh] or kansas[mesh] or kentucky[mesh] or louisiana[mesh] or maine[mesh] or maryland[mesh] or massachusetts[mesh] or michigan[mesh] or minnesota[mesh] or mississippi[mesh] or missouri[mesh] or montana[mesh] or nebraska[mesh] or nevada[mesh] or "new hampshire"[mesh] or "new jersey"[mesh] or "new mexico"[mesh] or "new york"[mesh] or "north carolina"[mesh] or "north dakota"[mesh] or ohio[mesh] or oklahoma[mesh] or oregon[mesh] or pennsylvania[mesh] or "rhode island"[mesh] or "south carolina"[mesh] or "south dakota"[mesh] or tennessee[mesh] or texas[mesh] or utah[mesh] or vermont[mesh] or virginia[mesh] or washington[mesh] or "west virginia"[mesh] or wisconsin[mesh] or wyoming[mesh] or "united states"[mesh] or "new york city"[mesh] or "district of columbia"[mesh] or "philadelphia"[mesh] or "baltimore"[mesh] or boston[mesh] or chicago[mesh] or "los angeles"[mesh] or "new orleans"[mesh] or "san francisco"[mesh] or "american samoa"[mesh] or guam[mesh] or "puerto rico"[mesh] or "united states virgin islands"[mesh] or alabama[ad] or alaska[ad] or arizona[ad] or arkansas[ad] or california[ad] or colorado[ad] or connecticut[ad] or delaware[ad] or florida[ad] or georgia[ad] or hawaii[ad] or idaho[ad] or illinois[ad] or indiana[ad] or iowa[ad] or kansas[ad] or kentucky[ad] or louisiana[ad] or maine[ad] or maryland[ad] or massachusetts[ad] or michigan[ad] or minnesota[ad] or mississippi[ad] or missouri[ad] or montana[ad] or nebraska[ad] or nevada[ad] or "new hampshire"[ad] or "new jersey"[ad] or "new mexico"[ad] or "new york"[ad] or "north carolina"[ad] or "north dakota"[ad] or ohio[ad] or oklahoma[ad] or oregon[ad] or pennsylvania[ad] or "rhode island"[ad] or "south carolina"[ad] or "south dakota"[ad] or tennessee[ad] or texas[ad] or utah[ad] or vermont[ad] or virginia[ad] or washington[ad] or "west virginia"[ad] or wisconsin[ad] or wyoming[ad] or guam[ad] or "puerto rico"[ad] or “american samoa”[ad] or “northern mariana islands”[ad] or "united states virgin islands"[ad] or "united states"[ad] or usa[ad])  #5) #1 AND #2 AND #3 AND #4  #6 ) #5 NOT (mice[tiab] OR mouse[tiab] OR rat[tiab] OR rats[tiab] OR rodent*[tiab] OR dog[tiab] OR dogs[tiab] OR pig[tiab] OR pigs[tiab] OR piglet*[tiab] OR swine[tiab] OR porcine*[tiab] OR animal*[tiab]) NOT ("Animals"[Mesh] NOT ("Animals"[Mesh] AND "Humans"[Mesh])) NOT (letter[ptyp] OR editorial[ptyp] OR comment[ptyp] OR news[ptyp] OR editorial[tiab] OR commentary[tiab] OR "retracted publication"[ptyp] OR "retraction of publication"[ptyp] OR "retraction of publication"[tiab] OR "retraction notice"[ti] OR "retracted publication"[tiab] OR "Published Erratum"[Publication Type] OR errata[tiab] OR erratum[tiab] OR corrigenda[tiab] OR corrigendum[tiab] OR protocol[ti] OR protocols[ti] OR "case report*"[tiab] OR "Case Reports" [Publication Type] OR "Congress"[Publication Type] OR "Consensus Development Conference"[Publication Type] OR editorial[tiab] OR commentary[tiab] OR “conference abstract*”[tiab] OR “conference proceeding*”[tiab]) AND (English[lang] OR Spanish[lang])  Date Filters: Search Update 2: 01/01/2022 – 01/25/2024 |

**Database:** Embase

**Platform:** Elsevier

**Date of Original Search:** April 28, 2021

**Date of Search Update 1**: May 18, 2022

**Date of Search Update 2**: Jan 25, 2024

**Original Search Limits:** Source: Embase; Language: English, Spanish; Publication year: 2009 – 2020

**Search Update 1 Limits:** Source: Embase; Language: English, Spanish; Publication date: 2021 – 2022

**Search Update 2 Limits:** Source: Embase; Language: English, Spanish; Publication date: 2022 – 2024

**Notes:** The same search strategy used in the original search was used for the search updates – only the date limits were changed. Use Advanced Search. Limit keywords to the title and abstract fields, and use the EMTREE controlled vocabulary and major EMTREE terms. Run the main search first and to these results add the limits for source, language, and publication year. Next, add the search strategies to exclude the animal studies and publication types not of interest. To these results, exclude the medline and pubmed-not-medline records, and then limit using Document Type to articles, review, and articles in press.

| **Search Strategy** |
| --- |
| #1) (disparit*:ti,ab OR underrepresent*:ti,ab OR "under represent*":ti,ab OR minority:ti,ab OR minorities:ti,ab OR "special population*":ti,ab OR "African American*":ti,ab OR black:ti,ab OR blacks:ti,ab OR Hispanic*:ti,ab OR Latino*:ti,ab OR Latina*:ti,ab OR "American Indian*":ti,ab OR "native American*":ti,ab OR "Alaska native*":ti,ab OR “Asian American*”:ti,ab OR "native Hawaiian*":ti,ab OR "pacific islander*":ti,ab OR guamanian*:ti,ab OR chamorro*:ti,ab OR samoan*:ti,ab OR "Mexican American*":ti,ab OR chicano*:ti,ab OR chicana*:ti,ab OR "Puerto Rican*":ti,ab OR "Cuban american*":ti,ab OR 'minority group'/exp OR 'Asian American'/exp OR 'African American'/exp OR 'Hispanic'/exp OR 'Mexican American'/exp OR 'Alaska Native'/exp OR 'American Indian'/exp OR 'Native Hawaiian'/exp OR 'American Samoan'/exp OR 'Puerto Rican'/exp OR 'Pacific Islander'/exp OR ‘ancestry group’/exp/mj)  #2) ("clinical research":ti,ab OR "clinical study":ti,ab OR "clinical studies":ti,ab OR "clinical trial*":ti,ab OR “controlled trial*”:ti,ab OR 'clinical study'/exp OR 'clinical research'/exp OR 'clinical trial'/exp OR 'randomized controlled trial'/exp OR 'controlled clinical trial'/exp) AND (participat*:ti,ab OR participation:ti,ab OR recruit:ti,ab OR recruits:ti,ab OR recruited:ti,ab OR recruiting:ti,ab OR recruitment*:ti,ab OR retain*:ti,ab OR retention:ti,ab OR enrol*:ti,ab OR enroll*:ti,ab OR underenrol*:ti,ab OR under-enrol*:ti,ab OR engagement*:ti,ab OR engage*:ti,ab OR engaging:ti,ab OR "patient selection":ti,ab OR "selecting patient*":ti,ab OR 'patient participation'/exp OR 'patient selection'/exp OR barrier*:ti OR facilitate*:ti)  #3) ("heart disease*":ti,ab OR cancer*:ti,ab OR neoplasm*:ti,ab OR carcinoma*:ti,ab OR stroke:ti,ab OR strokes:ti,ab OR "Cerebrovascular Accident*":ti,ab OR diabetes:ti,ab OR "Alzheimer disease*":ti,ab OR "alzheimers disease":ti,ab OR "Alzheimer dementia*":ti,ab OR "alzheimers dementia*":ti,ab OR pneumonia*:ti,ab OR influenza*:ti,ab OR "kidney disease*":ti,ab OR "chronic obstructive pulmonary disease*":ti,ab OR COPD:ti,ab OR "pulmonary hypertension":ti,ab OR asthma:ti,ab OR 'heart disease'/exp/mj OR 'asthma'/exp/mj OR 'chronic obstructive lung disease'/exp/mj OR 'pulmonary hypertension'/exp/mj OR 'diabetes mellitus'/exp/mj OR 'Alzheimer disease'/exp/mj OR 'cerebrovascular accident'/exp/mj OR 'malignant neoplasm'/exp/mj OR 'pneumonia'/exp/mj OR 'influenza'/exp/mj OR 'kidney disease'/exp/mj)  #4) ((ALABAMA:ti,ab OR ALASKA:ti,ab OR ARIZONA:ti,ab OR ARKANSAS:ti,ab OR CALIFORNIA:ti,ab OR COLORADO:ti,ab OR CONNECTICUT:ti,ab OR DELAWARE:ti,ab OR FLORIDA:ti,ab OR GEORGIA:ti,ab OR HAWAII:ti,ab OR IDAHO:ti,ab OR ILLINOIS:ti,ab OR INDIANA:ti,ab OR IOWA:ti,ab OR KANSAS:ti,ab OR KENTUCKY:ti,ab OR LOUISIANA:ti,ab OR MAINE:ti,ab OR MARYLAND:ti,ab OR MASSACHUSETTS:ti,ab OR MICHIGAN:ti,ab OR MINNESOTA:ti,ab OR MISSISSIPPI:ti,ab OR MISSOURI:ti,ab OR MONTANA:ti,ab OR NEBRASKA:ti,ab OR NEVADA:ti,ab OR "NEW HAMPSHIRE":ti,ab OR "NEW JERSEY":ti,ab OR "NEW MEXICO":ti,ab OR "NEW YORK":ti,ab OR "NORTH CAROLINA":ti,ab OR "NORTH DAKOTA":ti,ab OR OHIO:ti,ab OR OKLAHOMA:ti,ab OR OREGON:ti,ab OR PENNSYLVANIA:ti,ab OR "RHODE ISLAND":ti,ab OR "SOUTH CAROLINA":ti,ab OR "SOUTH DAKOTA":ti,ab OR TENNESSEE:ti,ab OR TEXAS:ti,ab OR UTAH:ti,ab OR VERMONT:ti,ab OR VIRGINIA:ti,ab OR WASHINGTON:ti,ab OR "WEST VIRGINIA":ti,ab OR WISCONSIN:ti,ab OR WYOMING:ti,ab OR GUAM:ti,ab OR "PUERTO RICO":ti,ab OR "VIRGIN ISLANDS":ti,ab OR USA:ti,ab OR "United States":ti,ab OR US:ti OR "New York City":ti,ab OR "Los Angeles":ti,ab OR Houston:ti,ab OR Dallas:ti,ab OR Miami:ti,ab OR Chicago:ti,ab OR Dallas:ti,ab OR Detroit:ti,ab OR Seattle:ti,ab OR "San Francisco":ti,ab OR Sacramento:ti,ab OR "San Diego":ti,ab OR Boston:ti,ab OR "Washington DC":ti,ab OR Baltimore:ti,ab OR Pittsburgh:ti,ab OR Philadelphia:ti,ab OR Denver:ti,ab OR Phoenix:ti,ab OR "New Orleans":ti,ab OR u.s.:ti OR u.s.a.:ti,ab OR American*:ti,ab OR "American Samoa":ti,ab OR "Northern Mariana Islands":ti,ab OR ALABAMA:de OR ALASKA:de OR ARIZONA:de OR ARKANSAS:de OR CALIFORNIA:de OR COLORADO:de OR CONNECTICUT:de OR DELAWARE:de OR FLORIDA:de OR GEORGIA:de OR HAWAII:de OR IDAHO:de OR ILLINOIS:de OR INDIANA:de OR IOWA:de OR KANSAS:de OR KENTUCKY:de OR LOUISIANA:de OR MAINE:de OR MARYLAND:de OR MASSACHUSETTS:de OR MICHIGAN:de OR MINNESOTA:de OR MISSISSIPPI:de OR MISSOURI:de OR MONTANA:de OR NEBRASKA:de OR NEVADA:de OR "NEW HAMPSHIRE":de OR "NEW JERSEY":de OR "NEW MEXICO":de OR "NEW YORK":de OR "NORTH CAROLINA":de OR "NORTH DAKOTA":de OR OHIO:de OR OKLAHOMA:de OR OREGON:de OR PENNSYLVANIA:de OR "RHODE ISLAND":de OR "SOUTH CAROLINA":de OR "SOUTH DAKOTA":de OR TENNESSEE:de OR TEXAS:de OR UTAH:de OR VERMONT:de OR VIRGINIA:de OR WASHINGTON:de OR "WEST VIRGINIA":de OR WISCONSIN:de OR WYOMING:de OR GUAM:de OR "PUERTO RICO":de OR "United states virgin islands":de OR "United States":de OR "New York City":de OR "District of Columbia":de OR "Philadelphia":de OR "Baltimore":de OR Boston:de OR Chicago:de OR "Los Angeles":de OR "New Orleans":de OR "San Francisco":de OR "American Samoa":de OR ALABAMA:ad OR ALASKA:ad OR ARIZONA:ad OR ARKANSAS:ad OR CALIFORNIA:ad OR COLORADO:ad OR CONNECTICUT:ad OR DELAWARE:ad OR FLORIDA:ad OR GEORGIA:ad OR HAWAII:ad OR IDAHO:ad OR ILLINOIS:ad OR INDIANA:ad OR IOWA:ad OR KANSAS:ad OR KENTUCKY:ad OR LOUISIANA:ad OR MAINE:ad OR MARYLAND:ad OR MASSACHUSETTS:ad OR MICHIGAN:ad OR MINNESOTA:ad OR MISSISSIPPI:ad OR MISSOURI:ad OR MONTANA:ad OR NEBRASKA:ad OR NEVADA:ad OR "NEW HAMPSHIRE":ad OR "NEW JERSEY":ad OR "NEW MEXICO":ad OR "NEW YORK":ad OR "NORTH CAROLINA":ad OR "NORTH DAKOTA":ad OR OHIO:ad OR OKLAHOMA:ad OR OREGON:ad OR PENNSYLVANIA:ad OR "RHODE ISLAND":ad OR "SOUTH CAROLINA":ad OR "SOUTH DAKOTA":ad OR TENNESSEE:ad OR TEXAS:ad OR UTAH:ad OR VERMONT:ad OR VIRGINIA:ad OR WASHINGTON:ad OR "WEST VIRGINIA":ad OR WISCONSIN:ad OR WYOMING:ad OR GUAM:ad OR "PUERTO RICO":ad OR "United states virgin islands":ad OR "United States":ad OR USA:ad)  #5) #1 AND #2 AND #3 AND #4  #6) #5 NOT (mice:ti,ab OR mouse:ti,ab OR rat:ti,ab OR rats:ti,ab OR dog:ti,ab OR dogs:ti,ab OR pig:ti,ab OR pigs:ti,ab OR piglet*:ti,ab OR swine:ti,ab OR porcine*:ti,ab OR rodent*:ti,ab OR animal*:ti,ab OR [animal cell]/lim OR [animal experiment]/lim OR [animal model]/lim OR [animal tissue]/lim) NOT ([animals]/lim NOT ([animals]/lim AND [humans]/lim)) NOT ([conference abstract]/lim OR [conference paper]/lim OR [conference review]/lim OR [editorial]/lim OR [erratum]/lim OR [letter]/lim OR [note]/lim OR [short survey]/lim OR 'editorial'/exp OR 'letter'/exp OR 'erratum'/exp OR 'retraction notice'/exp OR 'note'/exp OR 'short survey'/exp OR 'conference abstract'/exp OR 'conference paper'/exp OR “conference abstract*”:ti,ab OR “conference proceeding*”:ti,ab OR ‘consensus development’/exp OR corrigenda:ti,ab OR corrigendum:ti,ab OR erratum:ti,ab OR errata:ti,ab OR letter:ti,ab OR editorial:ti,ab OR commentary:ti,ab OR “retraction notice”:ti OR “retraction of publication”:ti,ab OR protocol:ti OR protocols:ti OR “case report*”:ti,ab OR 'case report'/exp) AND ([article]/lim OR [article in press]/lim OR [review]/lim) NOT [medline]/lim NOT [pubmed-not-medline]/lim) AND ([english]/lim OR [spanish]/lim)  Original Search Date filter: AND [2009-2020]/py)  Search Update 1 filter: AND [2020 - 2021]/py)  Search Update 2 filter: AND [2022 - 2024]/py) |

**Database:** Web of Science: Core Collection

**Platform:** Clarivate Analytics

**Date of Original Search:** April 29, 2021

**Date of Search Update 1**: May 18, 2022

**Date of Search Update 2**: Jan 25, 2024

**Limits:** Language: English, Spanish; Publication year: 2009 – 2020

**Search Update Limits:** Language: English, Spanish; Publication date: 2021 – 2022

**Search Update Limits:** Language: English, Spanish; Publication date: 2022 – 2024

**Notes:** The same search strategy used in the original search was used for the search updates – only the date limits were changed. Limit keywords to the Topic field (title, abstract, keywords, KeywordsPlus), but the geographical terms were limited to the Topic, City, Country, and State fields also. Run the main search first, and then to the combined search add the limits for publication year and language. Then to these results exclude the animal studies. To the final set of results, exclude the Document Types not of interest by using the filter from the search results page (exclude all but Article, Review, Early Access).

| **Search Strategy** |
| --- |
| #1) TS=((disparit* OR underrepresent* OR “under represent” OR “under represented” OR minority OR minorities OR “special population” OR “special populations” OR “ethnic group” OR “ethnic groups” OR “racial group” OR “racial groups” OR “African American” OR “African Americans” OR black OR blacks OR Hispanic* OR Latino* OR Latina* OR “American Indian” OR “American Indians” OR “native American” OR “native Americans” OR “Alaska native” OR “Alaska natives” OR “Asian American” OR “Asian Americans” OR “native Hawaiian” OR “native Hawaiians” OR “pacific islander” OR “pacific islanders” OR guamanian* OR chamorro* OR samoan* OR “Mexican American” OR “Mexican Americans” OR chicano* OR chicana* OR “Puerto Rican” OR “Puerto Ricans” OR “Cuban American” OR “Cuban Americans”)  #2) TS=(“clinical research” OR “clinical study” OR “clinical studies” OR “clinical trial” OR “clinical trials” OR “controlled trial” OR “controlled trials” OR “controlled clinical trial” OR “controlled clinical trials”) AND (underenrol* OR “under-enroll” OR “under-enrollment” OR “under-enrollments” OR “under-enrolls” OR “under-enrolled” OR “under-enrolling” OR participat* OR participation OR recruit OR recruits OR recruited OR recruiting OR recruitment* OR retain* OR retention OR enrol* OR enroll* OR engagement* OR engage* OR engaging OR “patient selection” OR “selecting patients” OR barrier* OR facilitate*)  #3) TS= (“heart disease” OR “heart diseases” OR cancer* OR neoplasm* OR carcinoma* OR stroke OR strokes OR “Cerebrovascular Accident” OR “cerebrovascular accidents” OR diabetes OR “Alzheimer disease” OR “Alzheimer diseases” OR “alzheimers disease” OR “alzheimers diseases” OR “Alzheimer dementia” OR “alzheimers dementia” OR pneumonia* OR influenza* OR “kidney disease” OR “kidney diseases” OR “chronic obstructive pulmonary disease” OR “chronic obstructive pulmonary diseases” OR COPD OR “pulmonary hypertension” OR asthma))  #4) TS=(alabama or alaska or arizona or arkansas or california or colorado or connecticut or delaware or florida or georgia or hawaii or idaho or illinois or indiana or iowa or kansas or kentucky or louisiana or maine or maryland or massachusetts or michigan or minnesota or mississippi or missouri or montana or nebraska or nevada or “new hampshire” or “new jersey” or “new mexico” or “new york” or “north carolina” or “north dakota” or ohio or oklahoma or oregon or pennsylvania or “rhode island” or “south carolina” or “south dakota” or tennessee or texas or utah or vermont or virginia or washington or “west virginia” or wisconsin or wyoming or guam or “puerto rico” or “virgin islands” or usa OR “United States” OR “New York City” OR “Los Angeles” OR Houston OR Dallas OR Miami OR Chicago OR Dallas OR Detroit OR Seattle OR “San Francisco” OR Sacramento OR “San Diego” OR Boston OR “Washington DC” OR Baltimore OR Pittsburgh OR Philadelphia OR Denver OR Phoenix OR “New Orleans” OR “U.S.A” OR “U.S.” OR US OR “American Samoa” OR “Northern Mariana Islands”) NOT dollar*) )  #5 PS=(alabama or alaska or arizona or arkansas or california or colorado or connecticut or delaware or florida or georgia or hawaii or idaho or illinois or indiana or iowa or kansas or kentucky or louisiana or maine or maryland or massachusetts or michigan or minnesota or mississippi or missouri or montana or nebraska or nevada or “new hampshire” or “new jersey” or “new mexico” or “new york” or “north carolina” or “north dakota” or ohio or oklahoma or oregon or pennsylvania or “rhode island” or “south carolina” or “south dakota” or tennessee or texas or utah or vermont or virginia or washington or “west virginia” or wisconsin or wyoming or guam or “puerto rico” or “virgin islands”)  #6 CU=(USA OR “United States” OR “U.S.A” OR “U.S.” OR US)  #7 CI=(“New York City” OR “Los Angeles” OR Houston OR Dallas OR Miami OR Chicago OR Dallas OR Detroit OR Seattle OR “San Francisco” OR Sacramento OR “San Diego” OR Boston OR “Washington DC” OR Baltimore OR Pittsburgh OR Philadelphia OR Denver OR Phoenix OR “New Orleans”)  #8 #4 OR #5 OR #6 OR #7    #9 #1 AND #2 AND #3 AND #8  #10 #9 NOT TS=((mice OR mouse OR rat OR rats OR dog OR dogs OR pig OR pigs OR piglet* OR swine OR porcine* OR rodent* OR animal*))  #11 #10 & Limit: Document Type include: Article, Review, Early Access  Date Filters:  **Original Search -** Publication date: and 2009 – 2020 (Publication Years)  **Update Search 1** - Publication date: and 2021 or 2022 (Publication Years)  **Update Search 2** - Publication date: and 2022 or 2023 or 2024 (Publication Years)  Refined by Languages: English, Spanish |

**Database:** CINAHL Plus

**Platform:** EBSCOhost

**Date of original search:** April 28, 2021

**Date of search update 1:** May 23, 2022

**Date of search update 2:** Jan 25, 2023

**Original Search Limits:** Language: English, Spanish; Publication year: 2009 – 2020

**Search Update 1 Limits:** Language: English, Spanish; Publication year: 2021 – 2022

**Search Update 2 Limits**: Language: English, Spanish; Publication year: 2022 – Jan 25, 2024

**Notes:** The same search strategy used in the original search was used for the search update – only the date limits were changed. Use Advanced Search. Use keywords and add corresponding CINAHL Subject Headings. Run the searches in the order specified and apply the limits as indicated to exclude animal studies and publication types not of interest.

| **Search Strategy** |
| --- |
| #1) disparit* OR MM "Healthcare Disparities" OR MM "Health Status Disparities" OR underrepresent* OR "under represent*" OR minority OR minorities OR "special population*" OR “ethnic group*” OR “racial group*” OR "African American*" OR TI black OR TI blacks OR Hispanic* OR Latino* OR Latina* OR "American Indian*" OR "native American*" OR "Alaska native*” OR “Asian American*” OR "native Hawaiian*" OR "pacific islander*" OR guamanian* OR chamorro* OR samoan* OR "Mexican American*" OR chicano* OR chicana* OR "Puerto Rican*" OR "Cuban american*" OR MH "Minority Groups" OR MH "Black Persons" OR MH "Ethnic Groups+" OR MH Asians OR MH "Hispanic Americans" OR "Mexican Americans" OR MH "Alaska Natives" OR MH "native americans" OR "native american indian*”  #2) "clinical research" OR "clinical study" OR "clinical studies" OR "clinical trial*" OR “controlled trial*” OR MH “clinical trials+” AND TI (underenrol* OR “under-enroll*” OR participat* OR participation OR recruit OR recruits OR recruited OR recruiting OR recruitment* OR retain* OR retention OR enrol* OR enroll* OR engagement* OR engage* OR engaging OR "patient selection" OR "selecting patient*”) OR MM “research subject recruitment” OR MM “research subject retention”  #3) ("heart disease*" OR MH "Heart Diseases" OR cancer* OR neoplasm* OR carcinoma* OR MH "Neoplasms+" OR stroke OR strokes OR MH "Stroke" OR diabetes OR MH "Diabetes Mellitus" OR "Alzheimer disease*" OR "alzheimers disease" OR "Alzheimer dementia*" OR "alzheimers dementia*" OR MH "Alzheimer’s Disease" OR pneumonia* OR MH "Pneumonia" OR influenza* OR MH "Influenza, Human” OR "kidney disease*" OR MH "Kidney Diseases" OR "chronic obstructive pulmonary disease*" OR COPD OR MH "Pulmonary Disease, Chronic Obstructive” OR "pulmonary hypertension" OR MH "Hypertension, Pulmonary" OR asthma OR MH "Asthma")  S4 - ALABAMA OR ALASKA OR ARIZONA OR ARKANSAS OR CALIFORNIA OR COLORADO OR CONNECTICUT OR DELAWARE OR FLORIDA OR GEORGIA OR HAWAII OR IDAHO OR ILLINOIS OR INDIANA OR IOWA OR KANSAS OR KENTUCKY OR LOUISIANA OR MAINE OR MARYLAND OR MASSACHUSETTS OR MICHIGAN OR MINNESOTA OR MISSISSIPPI OR MISSOURI OR MONTANA OR NEBRASKA OR NEVADA OR "NEW HAMPSHIRE" OR "NEW JERSEY" OR "NEW MEXICO" OR "NEW YORK" OR "NORTH CAROLINA" OR "NORTH DAKOTA" OR OHIO OR OKLAHOMA OR OREGON OR PENNSYLVANIA OR "RHODE ISLAND" OR "SOUTH CAROLINA" OR "SOUTH DAKOTA" OR TENNESSEE OR TEXAS OR UTAH OR VERMONT OR VIRGINIA OR WASHINGTON OR "WEST VIRGINIA" OR WISCONSIN OR WYOMING OR USA OR "United States" OR U.S. OR u.s.a. OR American* OR "New York City" OR "Los Angeles" OR Houston OR Dallas OR Miami OR Chicago OR Dallas OR Detroit OR Seattle OR "San Francisco" OR Sacramento OR "San Diego" OR Boston OR "Washington DC" OR Baltimore OR Pittsburgh OR Philadelphia OR Denver OR Phoenix OR "New Orleans" OR "American Samoa" OR "Northern Mariana Islands" OR GUAM OR "PUERTO RICO" OR "VIRGIN ISLANDS" OR MH “united states+” OR "New York City" OR "District of Columbia" OR "Philadelphia" OR "Baltimore" OR Boston OR Chicago OR "Los Angeles” OR "New Orleans" OR "San Francisco" OR "American Samoa" OR GUAM OR "PUERTO RICO" OR "United states virgin islands"  S5 - AF ALABAMA OR AF ALASKA OR AF ARIZONA OR AF ARKANSAS OR AF CALIFORNIA OR AF COLORADO OR AF CONNECTICUT OR AF DELAWARE OR AF FLORIDA OR AF GEORGIA OR AF HAWAII OR AF IDAHO OR AF ILLINOIS OR AF INDIANA OR AF IOWA OR AF KANSAS OR AF KENTUCKY OR AF LOUISIANA OR AF MAINE OR AF MARYLAND OR AF MASSACHUSETTS OR AF MICHIGAN OR AF MINNESOTA OR AF MISSISSIPPI OR AF MISSOURI OR AF MONTANA OR AF NEBRASKA OR AF NEVADA OR AF "NEW HAMPSHIRE" OR AF "NEW JERSEY" OR AF "NEW MEXICO" OR AF "NEW YORK" OR AF "NORTH CAROLINA" OR AF "NORTH DAKOTA" OR AF OHIO OR AF OKLAHOMA OR AF OREGON OR AF PENNSYLVANIA OR AF "RHODE ISLAND" OR AF "SOUTH CAROLINA" OR AF "SOUTH DAKOTA" OR AF TENNESSEE OR AF TEXAS OR AF UTAH OR AF VERMONT OR AF VIRGINIA OR AF WASHINGTON OR AF "WEST VIRGINIA" OR AF WISCONSIN OR AF WYOMING OR AF GUAM OR AF "PUERTO RICO" OR AF “American Samoa” OR AF “Northern Mariana Islands” OR AF "United states virgin islands" OR AF "United States" OR AF USA  #6) #4 OR #5  #7 ) #1 AND #2 and #3 and #6  #8) #7 NOT (mice OR mouse OR rat OR rats OR rodent* OR dog OR dogs OR pig OR pigs OR piglet* OR swine OR porcine* OR animal* OR MH animals+)  NOT (PT letter OR PT editorial OR comment OR MH news OR editorial OR PT commentary OR "retracted publication" OR "retraction of publication" OR "retraction of publication" OR "retraction notice" OR "retracted publication" OR MH “retracted publication” OR MH “retraction of publication” OR "Published Erratum" OR errata OR PT erratum OR corrigenda OR corrigendum OR PT protocol OR protocol* OR PT “case study” OR "case report*" OR MH "Case Reports" OR MH "Congresses and Conferences” OR "Consensus Development Conference" OR editorial OR PT commentary OR “conference abstract*” OR “conference proceeding*”)  **Original Search Limiters –** Publication Year – 2009–2021  **Search Update 1 Limiters**- Publication Year – 2021-2022  **Search Update 2 Limiters** – Publication Year – 2022-2024  **Language Limiters** – English, Spanish |
