# Supplementary material for: Recruitment and retention strategies for improving representation in clinical research: A meta-synthesis
Source: PLoS One. 2025 Jun 23;20(6):e0322796. doi: 10.1371/journal.pone.0322796 (PMC12184919; doi:10.1371/journal.pone.0322796)
Supplement: S4 Appendix — Quality assessment for each study included in the review. The table list all the studies that were included in the systematic review and the questions that were asked to assess whether the study partially met or fully met all criteria. (DOCX) [file pone.0322796.s005.docx]

**S4 Appendix. Quality Assessment.**

| Study | Is the role of the researcher clearly described? | Does the study indicate recruitment retention or both? | Does the study indicate specific chronic disease in inclusion criteria? | Is the source of whom is collecting the data mentioned or not mentioned? | Are the strategies made supported by sufficient evidence? | Met All Criteria |
| --- | --- | --- | --- | --- | --- | --- |
| Alvarado 2023 | Y | Y | Y | Y | Y | X |
| An 2023 | Y | Y | Y | Y | Y | X |
| Aranda 2023 | Y | Y | Y | N | Y |  |
| Crabbe 2023 | Y | Y | Y | Y | Y | X |
| Currier 2023 | Y | Y | Y | N | Y |  |
| Dance 2021 | Y | Y | Y | N | Y |  |
| Fink 2023 | Y | Y | Y | N | Y |  |
| Frierson 2019 | Y | Y | Y | Y | Y |  |
| Haynes-Maslow 2014 | Y | Y | Y | N | Y |  |
| Hartley-Brown 2024 | Y | Y | Y | N | Y |  |
| Hernandez 2021 | Y | Y | Y | Y | Y | X |
| Joseph 2009 | Y | Y | Y | Y | Y | X |
| Lincoln 2021 | Y | Y | Y | Y | Y | X |
| Legor 2023 | Y | Y | Y | N | Y |  |
| Medina 2023 | Y | Y | Y | N | Y |  |
| Mesa 2023 | Y | Y | Y | N | Y |  |
| Niranjan 2019* (training needs) | Y | Y | Y | N | Y |  |
| Niranjan 2021 (institutional influences) | Y | Y | Y | N | Y |  |
| Portacolone 2020 | Y | Y | Y | Y | Y | X |
| Regnante 2020 | Y | Y | Y | N | Y |  |
| Ridley-Merriweather 2022 | Y | Y | Y | Y | Y | X |
| Rivers 2019 | Y | Y | Y | Y | Y | X |
| Robinson 2020 | Y | Y | Y | Y | Y | X |
| Schatz 2023 | Y | Y | Y | N | Y |  |
| Stockdill 2023 | Y | Y | Y | Y | Y | X |
| TaPark 2023 | Y | Y | Y | Y | Y | X |
| Vickers 2023 | Y | Y | Y | Y | Y | X |
