# Supplementary material for: Recruitment and retention strategies for improving representation in clinical research: A meta-synthesis
Source: PLoS One. 2025 Jun 23;20(6):e0322796. doi: 10.1371/journal.pone.0322796 (PMC12184919; doi:10.1371/journal.pone.0322796)
Supplement: S5 Appendix — Data extracted from the primary research sources that were included in this systematic review. This details study characteristics including race/ethnicity, condition of interest, sex where noted, role in clinical research (e.g., participant, clinician (i.e., medical or research staff), community leader, etc.), geography of study (urban or rural), the name of the data extractors, date of data extraction, and the confirmation that the study was eligible for inclusion. (DOCX) [file pone.0322796.s006.docx]

**S5 Appendix. All Extracted Data from Included Studies.**
