# Supplementary material for: Recruitment and retention strategies for improving representation in clinical research: A meta-synthesis
Source: PLoS One. 2025 Jun 23;20(6):e0322796. doi: 10.1371/journal.pone.0322796 (PMC12184919; doi:10.1371/journal.pone.0322796)
Supplement: S2 Table — Inclusion criteria (study purpose – recruitment or retention, race and ethnicity, condition) are separated from other data extracted during the review. If an entry is missing, then the characteristic is unreported and/or not disaggregated. F = female, M = Male. SD = standard deviation. PI = principal investigator. Study partners = typically a family member or close friend. CBO Admin = community-based organization administrator. * Data in Niranjan references appear to be derived from the same Sources and therefore counted only once when totaling individuals reflected in this meta-synthesis. Quality assessment was conducted, where studies either met all or partially met criteria per on Critical Appraisal Skills Programme. Studies that partially met criteria remained included given their relevancy. The most common reason for partially met criteria was related to the sampling method. (DOCX) [file pone.0322796.s007.docx]

**S2 Table. Characteristics of included studies for meta-synthesis of evidence-based strategies (N=27).**

| **Study** | **Study Purpose** | | **Race and Ethnicity** | | **Condition of Interest (subtype if applicable)** | **Sex as Study Criteria** | **Role in**  **Clinical Research** | **Geography** | | **Met All Criteria** |  |
| --- | --- | --- | --- | --- | --- | --- | --- | --- | --- | --- | --- |
| Alvarado 2023 | Recruit | Latinx | | | Kidney Disease | Yes, Female, Male | Recruiters, Clinicians, Participants | Urban and Rural | | X |  |
| An 2023 | Recruit | Black/  African American | | | Cancer | Yes, Female, Male | Clinicians, Participants | Urban and Rural | X | | |
| Aranda 2023 | Recruit | Latinx | | | Alzheimer’s/  Dementia |  | Clinicians | Urban and Rural | |  |  |
| Crabbe 2023 | Recruit | Black/  African American | | | Alzheimer’s/  Dementia | Yes,  Female | Clinicians | Urban and Rural | | X |  |
| Currier 2023 | Recruit | Latinx | | | Cancer |  | Medical Center | Urban and Rural | |  |  |
| Dance 2021 | Recruit | Black/  African American | | | Cancer:  Lymphoma |  | Participants Unknown Unknown | | |  |  |
| Fink 2023 | Recruit/  Retain | POC | | | Cancer |  | Clinicians | Urban and Rural | |  |  |
| Frierson 2019 | Recruit | Black/  African American | | | Cancer: Breast | Yes, Female | Participants | Unknown | |  |  |
| Haynes-Maslow 2014 | Recruit | Black/  African American | | | Cancer: Breast, Cervical, Colon, Leg, Lung, Hodgkin’s, Skin, Non-Hodgkin’s, Ovarian | Yes, Female | Participants, Participant Networks | Urban and Rural | |  |  |
| Hartley-Brown 2024 | Recruit | POC | | | Cancer: Melanoma |  | Clinicians | Unknown | |  |  |
| Hernandez 2021 | Recruit | Black/  African American | | | Cancer: Bone, Breast, Lung, Prostate, Nasopharynx, Colon |  | Participants | Urban | | X |  |
| Joseph 2009 | Recruit | POC | | | Cancer: Breast | Yes,  Female | Clinicians | Urban | | X |  |
| Lincoln 2021 | Recruit | Black/  African American | | | Alzheimer’s/  Dementia |  | Participants | Urban | | X |  |
| Legor 2023 | Recruit | POC | | | Cancer | Yes, Female,  Male | Clinicians, Medical Center, Participants | Urban and Rural | |  |  |
| Medina 2023 | Recruit | POC | | | Cancer: Hematologic | Yes, Female,  Male | Clinicians, Medical Center | Urban and Rural | |  |  |
| Mesa 2023 | Recruit | POC | | | Cancer |  | Clinicians | Urban and Rural | |  |  |
| Niranjan 2019* (training needs) | Recruit/  Retain | POC | | | Cancer |  | Clinicians, Research Staff | Urban and Rural | |  |  |
| Niranjan 2021 (institutional influences) | Recruit | POC | | | Cancer |  | Clinicians, Research Staff | Urban and Rural | |  |  |
| Portacolone 2020 | Recruit | Black/  African American | | | Alzheimer’s/  Dementia |  | Participants, Participant Networks, Community Leader | Unknown | | X |  |
| Regnante 2020 | Recruit | POC | | Cancer | |  | Medical Center | Unknown | |  |  |
| Ridley-Merriweather 2022 | Recruit | Asian, Latinx | | Cancer: Breast | | Yes, Female | Participants | Unknown | | X |  |
| Rivers 2019 | Recruit | Black/  African American | | | Cancer | Yes, Female | Participants | Urban | | X |  |
| Robinson 2020 | Recruit | Black/  African American | | | Alzheimer’s/  Dementia | Yes, Female, Male | Participants,  Participant Networks | Urban and Rural | | X |  |
| Schatz 2023 | Recruit | POC | | | Cancer |  | Clinicians | Urban and Rural | |  |  |
| Stockdill 2023 | Recruit | Black/  African American | | | Heart Disease |  | Clinicians | Urban and Rural | | X |  |
| TaPark 2023 | Recruit | Asian, Native Hawaiian/ Pacific Islander | | | Alzheimer’s/  Dementia |  | Clinicians | Urban and Rural | | X |  |
| Vickers 2023 | Recruit | POC | | | Cancer |  | Clinicians | Urban and Rural | |  |  |
