# Supplementary material for: Recruitment and retention strategies for improving representation in clinical research: A meta-synthesis
Source: PLoS One. 2025 Jun 23;20(6):e0322796. doi: 10.1371/journal.pone.0322796 (PMC12184919; doi:10.1371/journal.pone.0322796)
Supplement: S3 Table — American Indian/Alaska Native are not listed because no studies included these populations. Niranjan 2019 and Fink 2023, focused on both recruitment and retention, were combined as recruitment for this table. Ridley-Merriweather 2022 focused on disaggregated strategies for both Asian and Latinos and TaPark 2023 focused on disaggregated strategies for both Asian and Native Hawaiian/Pacific Islander. There are 12 strategies, each listed in bold as headers. For each strategy, there are corresponding subcategories. In total, 27 studies were included in the analysis. The subcategories can be up to the maximum total for each strategy header. The subcategories may be counted multiple times for each theme. (DOCX) [file pone.0322796.s008.docx]

**S3 Table. Summary of recruitment strategies for groups less represented in clinical research.**

| **Strategy** | **Number of studies** | **Percent** | **Asian** | **Black/**  **African American** | **Native Hawaiian/Pacific Islander** | **Hispanic/ Latinx/** | **People of Color** |
| --- | --- | --- | --- | --- | --- | --- | --- |
| **Awareness** | **8** | **29.6%** | 0 | 5 | 0 | 0 | 3 |
| **Communication** | **13** | **48.1%** | 1 | 6 | 0 | 2 | 4 |
| Information- Sharing | 9 | 69.2% | 1 | 3 | 0 | 2 | 3 |
| Narrative Based | 4 | 30.7% | 1 | 2 | 0 | 1 | 0 |
| **Community-Based Participatory Research** | **17** | **62.9%** | 1 | 10 | 1 | 2 | 4 |
| Churches | 1 | 5.8% | 0 | 1 | 0 | 0 | 0 |
| Co-Design | 2 | 11.7% | 0 | 1 | 0 | 0 | 1 |
| Community Based  Organizations | 3 | 17.6% | 0 | 1 | 0 | 1 | 1 |
| Community  Engagement | 10 | 58.8% | 1 | 5 | 1 | 2 | 2 |
| Community External  Sites | 1 | 5.8% | 0 | 0 | 0 | 0 | 1 |
| Commitment | 3 | 15.7% | 0 | 1 | 0 | 1 | 1 |
| Community Leaders | 2 | 11.7% | 0 | 2 | 0 | 0 | 0 |
| Feedback | 3 | 17.6% | 0 | 2 | 0 | 0 | 1 |
| Partnerships | 9 | 52.9% | 1 | 4 | 1 | 2 | 2 |
| **Cultural Humility** | **10** | **37.0%** | 1 | 3 | 0 | 3 | 4 |
| Culturally Tailored | 6 | 60.0% | 0 | 2 | 0 | 2 | 2 |
| Shared Social Identity | 5 | 50.0% | 1 | 3 | 0 | 1 | 1 |
| **Education** | **12** | **44.4%** | 1 | 7 | 0 | 1 | 4 |
| Materials | 9 | 75.0% | 1 | 6 | 0 | 2 | 2 |
| Program | 5 | 41.6% | 1 | 3 | 0 | 1 | 0 |
| **Language** | **8** | **29.6%** | 2 | 2 | 1 | 2 | 4 |
| Accessible Verbiage | 7 | 87.5% | 1 | 2 | 0 | 1 | 4 |
| Multiple Languages | 7 | 87.5% | 2 | 2 | 1 | 2 | 2 |
| **Patient Navigation** | **7** | **25.9%** | 0 | 3 | 0 | 0 | 4 |
| **Patient Provider Relationship** | **9** | **33.3%** | 0 | 4 | 0 | 0 | 5 |
| **Social Networks** | **7** | **25.9%** | 2 | 2 | 1 | 2 | 2 |
| **Systems** | **6** | **22.2%** | 1 | 2 | 1 | 1 | 2 |
| Database | 5 | 71.4% | 1 | 1 | 1 | 1 | 1 |
| Policy/Procedure | 3 | 42.8% | 0 | 1 | 0 | 1 | 1 |
| **Technology** | **8** | **29.6%** | 1 | 4 | 0 | 1 | 2 |
| Social Media | 4 | 50.0% | 0 | 3 | 0 | 1 | 0 |
| Visuals | 4 | 50.0% | 0 | 4 | 0 | 0 | 0 |
| Information | 1 | 12.5% | 0 | 0 | 0 | 0 | 1 |
| **Training** | **5** | **18.5%** | 0 | 1 | 0 | 0 | 4 |
